# Systematic review of prognostic factors for poor outcome in people living with dementia that can be determined from primary care medical records

**DOI:** 10.1186/s12877-024-05389-0

**Published:** 2024-10-01

**Authors:** Michelle Marshall, Joanne L Jordan, Ram Bajpai, Danielle Nimmons, Tilli M Smith, Paul Campbell, Kelvin P Jordan

**Affiliations:** 1 School of Medicine, Keele University, Keele, ST5 5BG UK; 2 Research Department of Primary Care & Population Health, University College London, Royal Free Campus, Rowland Hill St, London, NW3 2PF UK; 3 Midlands Partnership University NHS Foundation Trust, St. George’s Hospital, Corporation Street, Stafford, ST16 3AG UK

**Keywords:** Dementia, Prognosis, Primary care, Systematic review, Care home admission, Cognitive decline, Palliative care

## Abstract

**Background:**

Dementia has a major impact on individuals, their families and caregivers, and wider society. Some individuals experience a faster decline of their function and health compared to others. The objective of this systematic review was to determine prognostic factors, measurable in primary care, for poor outcome in people living with dementia.

**Methods:**

Cohort studies set in the community or primary care, and examining prognostic factors for care home admission, cognitive decline, or palliative care were included. Databases were searched from inception to 17^th^ June 2022. Identified papers were screened, the risk of bias assessed using Quality in Prognostic Studies (QUIPS) tool, and data extracted by 2 reviewers, with disagreements resolved by consensus or a 3^rd^ reviewer. A narrative synthesis was undertaken, informed by GRADE, taking into consideration strength of association, risk of bias and precision of evidence. Patient and Public Involvement and Engagement (PPIE) and stakeholder input was obtained to prioritise factors for further investigation.

**Results:**

Searches identified 24,283 potentially relevant titles. After screening, 46 papers were included, 21 examined care home admission investigating 94 factors, 26 investigated cognitive decline as an outcome examining 60 factors, and 1 researched palliative care assessing 13 factors. 11 prognostic factors (older age, less deprived, living alone, white race, urban residence, worse baseline cognition, taking dementia medication, depression, psychosis, wandering, and caregiver’s desire for admission) were associated with an increased risk of care home admission and 4 prognostic factors (longer duration of dementia, agitation/aggression, psychosis, and hypercholesterolaemia) were associated with an increased risk of cognitive decline. PPIE and other stakeholders recommended further investigation of 22 additional potential prognostic factors.

**Conclusions:**

Identifying evidence for prognostic factors in dementia is challenging. Whilst several factors highlighted as of relevance by our stakeholder groups need further investigation, inequalities may exist in care home admission and there is evidence that several prognostic factors measurable in primary care could alert clinicians to risk of a faster progression.

**Registration:**

PROSPERO CRD42019111775.

**Supplementary Information:**

The online version contains supplementary material available at 10.1186/s12877-024-05389-0.

## Background

Dementia has a major impact on individuals, families, healthcare, and society and its impact is likely to increase with a growing ageing population. It is estimated that 982,000 people live with dementia in the UK, and this is projected to rise to 1.4 million by 2040 [1]. The National Institute for Health and Care Excellence (NICE) guidelines updated in 2023 [2] and more recently NHS England who produced a framework ‘The Well Pathway for Dementia’ [3] both highlighted the importance of supporting people to live well with dementia. Part of this is to prevent or delay consequences such as accelerated cognitive decline, early admission into formal care, and premature death. The key to why some individuals have faster progression is understanding better the factors that influence prognosis, which may then enable the ability to predict those who are likely to have a poorer trajectory following diagnosis. The identification of key evidence-based prognostic factors, measurable within UK primary care early in the course of dementia, could alert clinicians to patients with dementia who may be at risk of a worse trajectory and inform shared decisions on care and support between patients, carers, and clinicians. Modifiable prognostic factors could then be targeted at the earliest time point to offer the possibility of intervening to slow progression [4]. However, while a lot of research has been undertaken on the risk factors that predict the onset of dementia, less is understood about what increases the risk of poor prognosis after diagnosis [5]. Existing reviews of prognostic factors in dementia have focussed on a single outcome, a single prognostic factor, were conducted several years ago, or had a wide scope in population, outcomes, or prognostic factors of reduced relevance to UK primary care [6–9].

Primary care is at the forefront of the care of people living with dementia in the UK and a person’s primary care records may contain key information on their dementia health including prognostic factors. For example, markers of dementia-related health from routine primary care electronic health records have been identified as part of the Measurement of Dementia Disease Progression in Primary Care (MEDDIP) study [10, 11]. Findings from the MEDDIP study suggested recording of some of these markers early after diagnosis may be associated with poorer long-term outcomes. However, further evidence is needed on the independent prognostic value of factors potentially measurable in primary care. Therefore, the aim of this systematic review was to determine the evidence-base on prognostic factors for poor outcomes in people living with dementia that could be assessed in primary care medical records.

## Methods

To identify the evidence base for prognostic factors of poor outcomes in people living with dementia we conducted a systematic review and then reviewed the findings in collaboration with Patient and Public Involvement and Engagement (PPIE) members and stakeholders to assess their face validity and whether they could be assessed in a primary care setting.

The study is reported according to the preferred reporting items for systematic reviews and meta-analyses (PRISMA) guidelines [12], and was conducted following recommendations for systematic reviews of prognostic factor studies [13]. The study protocol was registered on PROSPERO (international prospective register of systematic reviews) before the searches were undertaken and is available online (CRD42019111775).

### Search strategy

An experienced information specialist (JJ) undertook searches using database specific subject headings (e.g. MeSH) and text words. Search terms included key terms for dementia (including terms for subtypes: Alzheimer’s, vascular dementia, Lewy body) and the outcomes of interest (care home admission, cognitive decline, palliative care). The full search strategy for Ovid Medline is presented in Supplementary Table 1.

The following nine databases (and database platforms) were searched from their inception to 17^th^ June 2022: MEDLINE (OVID); EMBASE (OVID); Emcare (OVID); CINAHL (EBSCO); AMED (OVID); PsycINFO (EBSCO); Ageline; Web of Science - Science Citation Index, Conference Abstracts; HMIC (OVID) - includes DoH and King’s Fund databases (OVID).

A sensitive search filter, adapted from filters evaluated by Glanville et al. [14] was used to identify observational studies of prognosis factors and prediction models. Reference lists of included studies, existing systematic reviews, and grey literature were reviewed to identify any further studies.

There were no restrictions on the search time frame or the language in which the paper was written.

### Inclusion criteria

#### Study type

Longitudinal cohort studies (prospective or historical) and electronic health care record studies were included if they included patients diagnosed with dementia. Study designs not suitable for analysis of prognostic factors (e.g., systematic reviews; randomised controlled trials; cross-sectional designs; case-control studies; case reports; and qualitative studies) as well as letters, commentaries and secondary reports of studies were excluded. If only a study abstract was available and not the full text, then they were excluded.

#### Participants/population

Studies were included of people with diagnosed dementia (all types including Alzheimer’s, vascular, Lewy body, frontotemporal, Parkinson’s) and the majority (more than 50%) of participants were living in the community to ensure the findings are relevant to primary care populations. Studies were excluded if they only included people with mild cognitive impairment rather than a diagnosis of dementia or if the focus was on identifying risk factors for onset of dementia or progression to a diagnosis of dementia.

#### Setting

The review included studies where patients living with dementia were recruited or observed in the community or primary care healthcare settings. Studies were excluded if they recruited >50% participants from care homes, hospital in-patients, outpatient departments, rehabilitation centres, or emergency care settings and therefore, were deemed to not be studying a community-based, primary care population.

#### Prognostic factors

Prognostic factors were included if the reviewers considered they may be recorded within a primary care setting. The following types of candidate prognostic factors were expected to have been previously examined: sociodemographic characteristics including potential markers of inequality (e.g., age, sex, education, deprivation, ethnicity, geographical region, rural/urban location); measures of clinical status (e.g. comorbidities, frailty, pharmacy, symptoms); quality of life; contextual factors (e.g., caregiver support, living situation); lifestyle factors (e.g., smoking). Other factors that had been identified by the PPIE group (e.g., behaviour change, changes in social interactions, having a power of attorney put in place) were also of particular interest. Studies examining genetics, biomarkers or other factors unlikely to be measured or recorded in primary care were excluded.

#### Outcomes

The following outcomes were examined as they were deemed to be key long-term outcomes for assessing disease progression:

(i) care home admission (first recorded post-dementia diagnosis)
(ii) cognitive decline (using a validated measure e.g. Mini Mental State Examination (MMSE), Addenbrooke’s Cognitive Examination (ACE)
(iii) palliative care including hospice admission (first recorded post-dementia diagnosis).

Mortality was not included, as a recent review had been undertaken examining this outcome [7].

To ensure factors were prognostic over the mid to long term rather than markers or predictors of immediate outcome, studies had to have a mean or median length of follow-up of one year or more for all outcomes. Studies using unvalidated measure of cognitive decline and those with people already receiving palliative care were excluded.

### Screening and data extraction

References identified in the database searches were downloaded into EndNote reference management software and duplicates removed. Unique articles were uploaded to Rayyan online systematic reviewing software and titles were screened by a single reviewer (MM, JJ, DN, PC, KJ), due to the large number of references, to exclude articles where it was clear titles did not match the review inclusion criteria. A sample of 10% of titles were double screened, and if agreement was less than 95%, an additional proportion was screened by a third person.

Articles not excluded in the initial screening or if there was uncertainty from the title were uploaded to Covidence systematic review software for the further screening. Abstracts and the full texts of articles were independently screened by two reviewers (MM, KJ). Any disagreements were resolved by consensus, and if needed, in discussion with a third reviewer (DN).

Data extraction was completed independently by two out of five reviewers (MM, JJ, RB, TS, KJ) with disagreements resolved by consensus and if necessary, in discussion with a third reviewer. A standardised data extraction form using MS Excel was used to collect the following data for analysis: study population and setting; age and sex distribution; type of dementia; follow-up duration; sample size; retention rate; definition of prognostic factor; timing of prognostic factor; definition of outcome; covariates used in the analysis; unadjusted and adjusted estimates of the association between a candidate prognostic factor and an outcome.

### Quality assessment

The potential risk of bias in the included studies was assessed using Quality in Prognostic Studies (QUIPS) tool [15] with assessments recorded in MS Excel. At least two review authors (MM, JJ, RB, KJ, TS) independently graded risk of bias (unclear, high, or low risk of bias) for each domain of the QUIPS tool for each study. These assessments were then compared within each pair of reviewers, with disagreements resolved by discussion or by third reviewer.

### Synthesis

A narrative synthesis of the available evidence on potential factors in a primary care setting that affect the course of dementia was undertaken. Where possible factors were plotted in forest plots to help visualise and identify similarities and differences between the studies in estimates of association with outcomes. Synthesis took account of the evidence based on direction of effect (strength of the association), potential risk of bias, consistency, directness and precision for individual factors using a GRADE approach [16, 17]. This approach initially considered the group of studies for a potential prognostic factor as high-level evidence, and was reduced in level (to moderate, low, or very low) using pre-determined criteria (Supplementary Table 2).

Where there were three or more studies that examined the same factor for the same outcome, a random effects meta-analysis to pool adjusted estimates of association was also planned where there was clinical and study design homogeneity (same candidate prognostic factor and outcome). Statistical heterogeneity across studies was assessed by visual inspection of forest plots, alongside I^2^ statistics where possible, which provided quantitative information on between-study variability [13]. Where formal meta-analyses were not possible, potential prognostic factors following similar definitions were grouped together as much as possible to identify trends in the direction of effect and consistency of results through visual inspection of forest plots without pooling.

Based on the findings of the review, the GRADE assessment and forest plots, factors identified in the review were classified as:

- prognostic, i.e. associated with increased risk of poor outcome,
- not prognostic, i.e. not associated with increased risk of poor outcome,
- having inconsistent evidence with studies both for and against being prognostic, or
- having too limited evidence, such as one study, or all studies with high risk of bias or small sample size.

### Public and Patient Involvement (PPIE) and stakeholder involvement

Two meetings were held with members of the Keele Research User Group (RUG) who had experience of being caregivers of persons living with dementia. The aim of the meetings was to obtain their views, experience, and opinions regarding factors that may predict a poor outcome.

In the first meeting, the methods and aims of the systematic review were presented to five members. They were asked to consider what factors they felt might indicate or predict that a person’s dementia was progressing and was more likely to have poor outcome (for example, care home admission, need palliative care, death). To help stimulate ideas they were asked to consider factors under several different headings that included: the person living with dementia (e.g., symptoms); their medical care and treatments (e.g., medications); relationships and social aspects (e.g., difficulties in social settings); support and organisation of social care (e.g., needing more help); and health inequalities (e.g., deprivation). Factors suggested were considered to ensure our search strategy and selection criteria were appropriate so all possible prognostic factors would be identified.

In the second PPIE meeting the five attending members (two of whom had also attended the first meeting) reviewed the factors that had been suggested as important at the first meeting. The results of the review were presented and factors that had been identified as prognostic in the systematic review were compared with those suggested at the first meeting and discussed, and the group had the opportunity to suggest additional factors. The members also commented on the face validity of the results of the review from their experiences, and whether they would be factors that would be discussed with a general practitioner or practice nurse.

Following this, the evidence for the prognostic factors from the review and the feedback from the PPIE group were presented to a group of stakeholders that consisted of two general practitioners, an expert in research using health records, and an expert in dementia research. The group commented on the face validity of the factors identified in the review, proposed any factors they felt were missing, discussed the suggestions and comments made by the PPIE members, and highlighted any factors they felt it would not be feasible to be captured during primary care consultations and would not be coded in primary care electronic health records.

From the findings of the review, and the PPIE and stakeholder meetings a final list of prognostic factors of poor outcomes in people living with dementia and a list of factors that should be investigated further were derived. Factors felt to be unlikely to be recorded in primary care by the PPIE and stakeholder groups were excluded.

## Results

### Systematic review

**Fig. 1:**
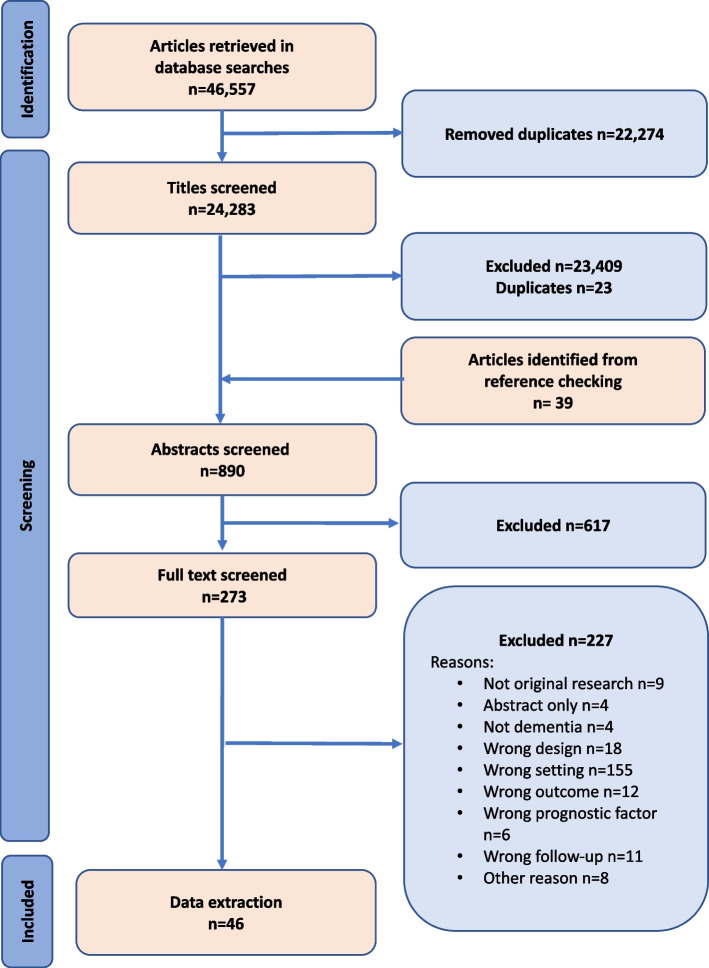
PRISMA 2020 flow diagram presenting study selection

The searches of the nine databases retrieved 46,557 references with a further 39 references identified from checking reference lists of the included studies and other sources. After removal of duplicates and screening 24,283 titles and abstracts, 273 full text articles were obtained. Full text screening reduced the number included in the review to 46 studies (Fig. 1).

### Studies

A summary of the 46 included studies is provided in Supplementary Table 3 with the full references given in Supplementary Table 4. Of these included studies, 21 examined care home admission including 294,896 individuals, 26 studies examined cognitive decline as an outcome in a total of 17,116 individuals, and one study examined palliative care including 30,463 individuals. Two studies looked at both care home admission and cognitive decline. The majority of studies were based in Europe (*n*=24) and North America (*n*=19) with two studies based in Oceania and one in South Asia. Thirty-seven studies used data from cohort studies and nine used medical record data.

### Risk of bias

A pragmatic approach was taken towards the risk of bias. A number of studies had individual items that were deemed at high risk of bias, but the summary decisions for each domain were assessed based on the impact this might have on the data collected and analysed. For example, if a study had little loss to follow-up, then the importance of methods for collecting or analysing data on dropouts was considered to be less important, thus such a study was not excessively penalised in the summary score across the study attrition domain.

Overall, 25 studies had at least one domain that was judged as being of high risk of bias and were hence considered to have a high overall risk of bias, 19 studies had all domains that were judged to be at moderate or low risk and thought to have a moderate overall risk of bias, and two studies had low risk of bias across all domains and were therefore deemed to be at low overall risk of bias (Supplementary Table 5). The main area of concern was in the attrition domain where 19 studies were judged to have a high risk of bias. By contrast, no study had a high risk of bias in the outcome domain.

### Strength of the evidence

The strength of evidence, based on the GRADE criteria, was assessed for each candidate prognostic factor for the outcomes of care home admission and cognitive decline and an overall judgement was agreed between the reviewers. For care home admission, 10 factors were considered to have high evidence, 52 moderate evidence and 32 low evidence (for full details see Supplementary Table 6). For cognitive decline, none of the factors were found to have high evidence, there was moderate evidence for 13 factors with the remaining 47 deemed to have low evidence (for full details see Supplementary Table 7). As there was only one study that examined the outcome of palliative care, the evidence was deemed to be limited and a GRADE assessment was not undertaken.

### Prognostic factors: care home admission

**Fig. 2:**
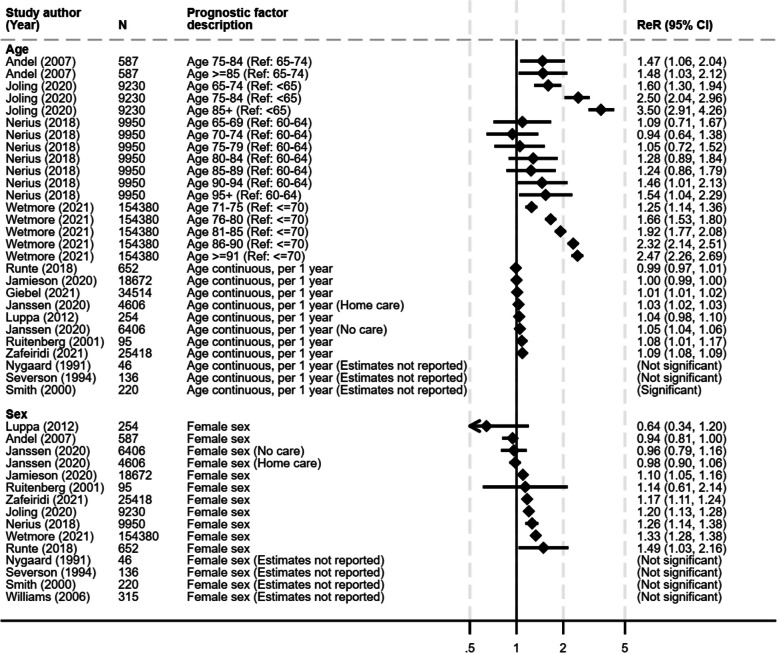
Forest plots of age and sex and their association with the care home admission. ReR, relative risk; CI, confidence interval; N, study sample size

**Fig. 3:**
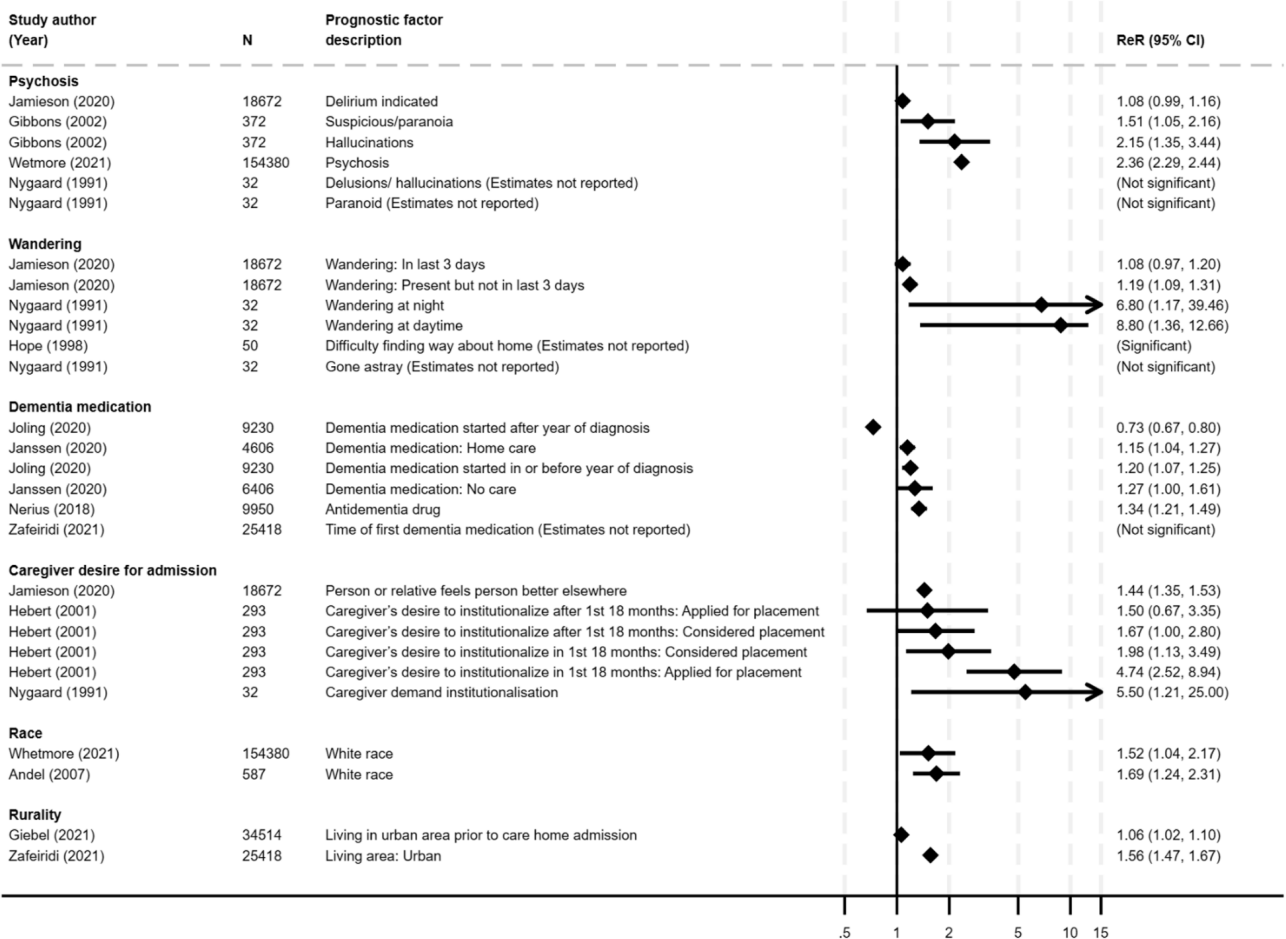
Forest plots of six prognostic factors and their association with care home admission. ReR, relative risk; CI, confidence interval; N, study sample size

A total of 94 candidate prognostic factors for care home admission had been investigated. The outcome of care home admission was examined consistently as a binary outcome (being admitted or not being admitted over the follow-up period) however, there were still variations in the definitions and reference categories used for the factors. Also, some studies only reported whether a factor was statistically significant or not with no estimate presented. Due to this heterogeneity, it was not possible to undertake any meta-analyses. For factors with similar definitions across studies, visualisation of trends in the direction of effect and consistency of results were examined using forest plots. Figures 2 and 3 show example forest plots for factors associated with an increased risk of care home admission and for sex where inconsistent evidence was found.

Overall, of the 94 factors investigated 11 were independently associated with an increased risk of care home admission, these were:

- older age,
- less deprived,
- living alone,
- white race,
- urban residence,
- worse baseline cognition,
- taking dementia medication,
- depression,
- psychosis,
- wandering,
- caregiver’s desire for admission),

Of the remaining factors identified, 15 showed no association with an increased risk of care home admission, for 18 the findings were inconsistent, and 50 factors had limited evidence investigated by only a single study (Table 1).

**Table 1:** Summary of potential prognostic factors for each outcome

**Care home admission** (***n*=94)**			
**Associated with an increased risk** ^a^ ***n*=11**	**Not associated with an increased risk **^b^ ***n*=15**	**Inconsistent evidence** ^c^***n*=18**	**Limited evidence **^d^ ***n*=50**
Older age Less deprived Living alone White race Urban residence Worse cognition at baseline Taking dementia medication Depression Psychosis/psychotic symptoms Wandering Caregiver desire for admission	BMI/weight Self-reported general health Aggression Agitation Mental health (composite) Cancer Respiratory disease Musculoskeletal disease Polypharmacy Bowel incontinence Hearing loss Urinary incontinence Visual impairment Caregiver mental health Caregiver residence	Sex Education Marital status Activities of daily living Mobility Type of dementia Behaviour change Hospitalisation No. of comorbidities Cardiovascular disease Cerebrovascular disease (inc stroke/TIA) Diabetes Frailty Currently receives care Caregiver age Caregiver sex Caregiver physical health Caregiver relationship	Alcohol Smoking Geographical region Income Migrant status Type of residence (unsupported/ retirement/assisted) Falls/gait problems Inactivity Safety concerns Lonely Social withdrawal Difficulties communicating Difficulty understanding Duration of dementia Anxiety Difficulty going to bed Fearful Orientation problems at home Inappropriate urination Irritable/easy to anger Apathy Major stress Making accusations Night-time activity Sleep disorders/disturbance Tearful No. of doctor visits Hypercholesterolaemia Hypertension Liver disease Peptic ulcer disease Renal disease Antipsychotic medication Painkillers Dyspnoea Eating problems Extrapyramidal signs Fatigue Caregiver burden/impact Caregiver quality of support Duration of care Size of support network Caregiver family conflict Caregiver guilt Caregiver employed Caregiver not enough time for self Caregiver loss of hobbies Caregiver sleep disturbance Caregiver main source of income Caregiver difficulty taking responsibility
**Cognitive decline** (***n*=60)**			
**Associated with an increased risk** ^a^ ***n*=4**	**Not associated with an increased risk **^b^ ***n*=6**	**Inconsistent evidence **^c^ ***n*=8**	**Limited evidence **^d^ ***n*=42**
Longer duration of dementia Agitation/aggression Psychosis/psychotic symptoms Hypercholesterolaemia	Self-reported general health No. of comorbidities Hypertension Cerebrovascular disease (inc stroke/TIA) Depression/anxiety/ irritability Smoking	Age Sex Education Cardiovascular disease Diabetes Antihypertensive medication Cognition at baseline Type of dementia	BMI/weight Frailty Literacy Living situation (alone/partner/family/ caregiver) Race Occupation (ever) Urban residence Activity disturbance Activities of daily living Difficulty dressing Falls/gait problems Gait speed Inactivity Difficulty with money Social network Social withdrawal Family history of dementia No. of cognitive activities Time from onset to diagnosis Apathy Complaining Emotionally liable Fearful Wandering Paces Hoards things No. of behaviour symptoms Severe neuropsychiatric symptoms Sleep disorders/disturbance Tearful Musculoskeletal disease Anticholinergic medication Antidepressant medication No. of antipsychotic medications Antipsychotic medication Diuretic medication NSAID medication Sedative medication Hypertension or stroke (assessed jointly) Extrapyramidal signs Hearing impairment Visual impairment

*BMI* Body mass Index, *NSAID* Non-Steroidal Anti-Inflammatory Drugs

^a^Increased risk: At least moderate evidence in GRADE assessment AND either consistent positive association in ≥2 studies OR low evidence and consistent positive association in ≥3 studies OR 1 study and positive association and low RoB

^b^No increased risk: At least moderate evidence in GRADE assessment AND either consistent negative or no association in ≥2 studies OR low evidence and consistent negative or no association in ≥3 studies OR 1 study and negative or no association and low RoB

^c^Inconsistent: ≥2 studies AND inconsistent findings which does not fulfil "Increased risk" or "No increased risk" criteria

^d^Limited evidence: Does not fulfil 3 other categories e.g. one study with moderate or high RoB, or low evidence in GRADE assessment in only 2 studies

### Prognostic factors: cognitive decline

A total of 60 candidate prognostic factors for cognitive decline were evaluated. A number of different assessments of cognitive decline had been undertaken including the Mini Mental State Examination (MMSE), Addenbrooke’s Cognitive Examination- revised (ACE-R), Clinical Dementia Rating (CDR), Cambridge Cognitive Examination (CAMCOG), Dementia Rating Scale (DRS), Modified Mini Mental State examination (3MS), and various individual and composite cognitive function assessments. In addition, there were variations in how the outcome was measured (binary, time to event, continuous), and in the definitions of factors, some studies also only reported whether a factor was statistically significant or not with no estimate presented. This heterogeneity meant that it was not possible to produce summary forest plots or undertake any meta-analyses.

Of the 60 candidate prognostic factors investigated, the presence of four were independently associated with an increased risk of cognitive decline (Table 1). These were:

- longer duration of dementia illness,
- agitation/aggression,
- psychosis,
- hypercholesterolaemia.

Presence of six factors did not increase risk of cognitive decline:

- worse self-reported general health,
- higher number of comorbidities,
- hypertension,
- cerebrovascular disease (stroke, transient ischaemic attacks),
- depression/anxiety/irritability,
- smoking.

A further eight factors were found to have inconsistent evidence, and the remaining 42 factors had evidence that was too limited for conclusions to be made (Table 1).

### Prognostic factors: palliative care

A total of 13 candidate prognostic factors from one study were examined for the outcome of palliative care. Each factor was a domain (e.g., cognitive function, safety, daily functioning) and had a number of associated markers (e.g., cognitive decline, memory loss, confusion, aphasia in the domain of cognitive function) attributed to it. As only a single study examined this outcome all factors were determined to have limited evidence.

### PPIE and stakeholder meeting outcomes

In the first PPIE meeting prior to the systematic review, 27 factors that might indicate or predict that a person living with dementia was progressing and was more likely to have poorer outcome (care home admission, need palliative care) were suggested. In the second PPIE meeting after the review the group reviewed the factors that had been suggested as important in the first meeting and a further eight factors were put forward by the group. The factors that had been assessed within studies included in the systematic review were then compared with those suggested by the PPIE group and 17 factors had been previously researched, leaving 18 factors identified by the group as potentially important but with lack of any research evidence (Table 2).

**Table 2:** PPIE and stakeholders’ views on factors they felt were missing from the review and factors identified in the review they felt would be difficult to assess in primary care electronic health records

PPIE additional factors suggested (*n*=18)	Stakeholder additional factors suggested (*n*=11)	Factors in review or identified by caregivers/stakeholders that would be difficult to assess in primary care (*n*=34)
Advocacy, people to support/fight for the person living with dementia Attention seeking Avoiding/covering up difficulties with cognitive testingBereavementBuild-up of number of factors Caregiver information received about dementia Change in living environment/ residence Change in monitoring own health Continuity of care within primary care Difficulty keeping up with household tasks Distance from relatives/caregivers Falling for scams Needing help with finances Physical fighting/violence^a^ Power of attorney Resisting support Time to diagnosis When factors developed in course of dementia	Bereavement Build-up of number of factors Change in living environment/residence Continuity of care within primary care Dietary supplements Disinhibition Increase in number of GP home visits Oral health (inc. oral thrush) Power of attorney Referral for imaging When factors developed in course of dementia	Activities of daily living Advocacy, people to support/fight for the person living with dementia Attention seeking Avoiding/covering up difficulties with cognitive testing Caregiver age, Caregiver behaviour change Caregiver desire for admission, Caregiver information received about dementia, Caregiver physical health, Caregiver quality of support Caregiver relationship, Caregiver sex Change in living environment/ residence Change in monitoring own health Complaining Difficulty keeping up with household tasks Disinhibition Distance from relatives/caregivers Education Emotionally liable Falling for scams Family history of dementia Inappropriate urination Income Literacy (limited to illiterate) Making accusations Marital status Migrant status Needing help with finances Occupation (limited to certain ones that are risky or require health assessment) Resisting support Socially withdrawal Time to diagnosis Type of residence (unsupported/ retirement/assisted)

^a^Defined by stakeholders as included in aggression

The stakeholders group reviewed the candidate prognostic factors identified in the review and proposed 11 additional factors they felt were missing (Table 2). Six of these factors were also suggested by the PPIE group.

The PPIE and stakeholder members also commented on the face validity of the results of the review, whether factors would be discussed with a general practitioner or practice nurse during a consultation, and whether they would be captured and coded in primary care electronic health medical records. Thirty-four factors from the review or PPIE/stakeholders meeting across the three outcomes were identified as being difficult to discuss in consultations or capture in primary care records (Table 2).

There were 22 additional factors recommended for further investigation following the PPIE and stakeholder meetings (Table 3). These included bowel and urinary incontinence which were not associated with an increased risk in the review, but PPIE and stakeholders considered that as the review focused on a minimum of twelve-month follow-up, these factors may indicate a reason or increased risk for more imminent care home admission depending on the current level of care the person has*.* These two groups were also keen to investigate the impact of comorbidity burden despite general lack of evidence of its prognostic value in the review. Caregiver’s desire for care home admission was felt implausible to measure in primary care, and would not be recorded, hence it has been excluded from the evidence-based prognostic factors.

**Table 3:** Summary list of primary care prognostic factors and factors needing further investigation for outcomes of care home admission and cognitive decline

Category	Evidence-based prognostic factors	Factors recommended for further investigation	Evidence from the review and/or from PPIE/Stakeholder
Sociodemographic	Older age Less deprived Living alone Race – white Urban residence		Increased risk CHA; Inconsistent CD Increased risk CHA Increased risk CHA; Limited CD Increased risk CHA; Limited CD Increased risk CHA; Limited CD
		Sex	Inconsistent CHA and CD
General health & lifestyle^a^			
Functional status		Mobility	Inconsistent CHA
Social^a^			
Dementia characteristics	Cognition at baseline Taking dementia medication Duration of dementia		Increased risk CHA; Inconsistent CD Increased risk CHA Increased risk CD; Limited CHA
		Type of dementia	Inconsistent CHA and CD
Neuropsychiatric	Depression Agitation/Aggression^a^ Psychosis Wandering		Increased risk CHA; No increased risk CD Increased risk CD; No increased risk CHA Increased risk CHA and CD Increased risk CHA, Limited CD
		Bereavement	PPIE/Stakeholder
Symptoms		Bowel Incontinence Urinary Incontinence	No increased risk CHA; PPIE/Stakeholder No increased risk CHA; PPIE/Stakeholder
Comorbidities	Hypercholesterolaemia		Increased risk CD; Limited CHA
		No. of comorbidities Hospitalisation Increase in numberof GP home visits Cardiovascular disease Cerebrovascular disease Diabetes Frailty Oral health (inc. oral thrush)	No increased risk CHA and CD; PPIE/Stakeholder Inconsistent CHA PPIE/Stakeholder Inconsistent CHA and CD No increased risk CD; Inconsistent CHA Inconsistent CHA and CD Inconsistent CHA; Limited CD PPIE/Stakeholder
Medications		Antihypertensive medication Dietary supplements	Inconsistent CD PPIE/Stakeholder
Care & support		Currently receiving care Continuity of care within primary care Power of attorney	Inconsistent CHA PPIE/Stakeholder PPIE/Stakeholder
Other		Referral for imaging Build-up of number of factors When factors developed in the course of dementia	PPIE/Stakeholder PPIE/Stakeholder PPIE/Stakeholder

*CHA* Care home admission outcome, *CD* Cognitive decline outcome, PPIE/Stakeholder, Factor thought to be important to pursue further by PPIE and/or stakeholder group

^a^No factors identified in these categories

A summary including the final list of prognostic factors of poor outcomes in people living with dementia from the findings of the review, as well as factors suggested by the PPIE and stakeholder group meetings is presented in Table 3.

## Discussion

This systematic review examined the evidence-base for factors that are routinely recorded or easily measurable in primary care and are prognostic for care home admission, cognitive decline, or palliative care. We found a large number of studies had examined care home admission and decline in cognition as outcomes but only one had examined referral to palliative care. The risk of bias was moderate to high for the majority of studies and only 10 factors (all for the outcome of care home admission) had high GRADE evidence. Overall, 11 factors were independently associated with an increased risk of care home admission and 4 factors were independently associated with an increased risk of cognitive decline. Following discussion with the PPIE and stakeholders groups, one of these factors (caregiver desire for admission) was felt implausible to be measured in primary care, and a further 22 were recommended for further research.

### Comparison to other studies

Older age, white race, and baseline cognition were associated with care home admission, and neuropsychiatric symptoms with care home admission and cognitive decline. These findings are consistent with previous reviews that have examined long term care placement and mortality outcomes for people living with dementia [6, 8, 9]. Neuropsychiatric factors (including psychosis, depression, agitation, aggression, and wandering), in particular, are strong prognostic factors for poorer outcomes. A previous review of risk factors for progression of Alzheimer’s disease found 12 studies which examined prognostic factors, with higher education associated with faster cognitive decline [18]. Education had inconsistent evidence in our review for both care home and cognitive function outcomes and was not recommended by our PPIE or stakeholder groups for further investigation. However, lower deprivation which is likely to be associated with higher education levels was a strong risk factor for care home admission. Some of the sociodemographic factors increasing risk of care home admission, such as lower levels of deprivation, white race, and urban residence, may reflect improved access and availability of care homes and may indicate inequalities in dementia care [19, 20].

Inconsistent associations or limited evidence was found for male sex, diabetes, smoking, and cardiovascular disease which have previously been found to be prognostic factors for earlier mortality in dementia [6, 8]. This may reflect different predictors for mortality or more broader definitions of populations, and particularly the setting, included in those reviews. It is possible that prognostic factors for people living with dementia drawn from secondary care settings or, for cognitive decline, from formal care settings will differ given they are likely to be associated with greater dementia severity. A number of caregiver prognostic factors have been investigated for the outcome of care home admission. The burden on the caregivers increases as dementia progresses, but the trajectory of this increased burden is highly variable [21]. In the current review we found a moderate or high level of evidence only for the caregiver’s desire for admission but this factor will overarch a whole range of factors including the caregivers’ situation, health, and associated burden [21].

We found only one study which had examined potential prognostic factors for palliative care (from a team that included authors of this review). This may reflect wide variation in definitions and measures been used for end-of-life care for people living with dementia [22] and evidence that people living with dementia have been found to be less likely to receive palliative care [23].

### Implications and future research

Identifying prognostic factors that indicate faster disease progression in primary care is important as it could help clinicians such as GPs identify the individuals diagnosed with dementia who may be at higher risk of a poorer prognosis. This would help clinicians better plan individualised care for these individuals and inform shared decisions on care and support between patients, family members, carers, and clinicians. It could also help healthcare policymakers and providers plan resources at the population level and may improve the efficiency of intervention studies, which currently rely on intensive and costly follow-up assessments and long-term outcomes such as mortality.

Further studies are required to investigate a number of factors. There are those that were highlighted by our PPIE and stakeholder groups as potentially being important markers of disease progression which have not been investigated sufficiently, or at all, in terms of their potential role in prognosis. Additionally, we identified some factors as potential inequalities in access to care homes which included deprivation, race, and rurality, and research is needed to explore the extent and impact of these on the care of people living with dementia. Further to this, given that the risk of care home admission and cognitive decline is likely to be multifactorial, the prognostic factors that we found to be associated with increased risk of poorer outcomes need to be investigated in interaction with markers of health inequalities and the new factors identified by PPIE and stakeholder to determine collectively factors that best indicates faster dementia progression.

### Strengths and limitations

This was an extensive systematic review which investigated 116 different potential prognostic factors, focussing on those which are likely to be easily measurable and/or recorded in primary care where most people living with dementia are managed. PPIE and stakeholder input was an important part of this study. Findings from the review were discussed with carers of people living with dementia and an expert stakeholder group and many factors considered by the PPIE group or by the stakeholders to be important based on their experience had not been examined in previous research studies.

Despite a rigorous search strategy and approaches to the review, and input from our PPIE and stakeholder groups, it is possible that some important factors may not have been identified. Many candidate prognostic factors were investigated in single studies. Measurement tools and definitions of cognitive decline varied making it harder to compare evidence for factors for this outcome than for care home admission where a binary or time to event outcome was used. Inconsistent evidence related to prognostic factors for cognitive decline may also be attributed to different start points for studies. A baseline measure of cognitive function measured after diagnosis may already have been influenced by a prognostic factor, and no association with change in cognition then identified. We identified several studies where a factor was positively associated with a baseline measure of cognition, but negatively or not associated with its change over time. There was also variation in the method of assessment, definition, and categorisation of potential factors so that pooling by meta-analysis was not possible, and whilst we used forest plots as a graphical aid to interpret the overall evidence, this excluded studies which did not report estimates of association. In addition, some studies grouped several factors together while other studies examined them separately which has meant that there are some variations in factors for the different outcomes. For example, we were able to examine aggression and agitation separately for care home admission but could only examine them in combination for cognitive decline.

## Conclusions

Uncovering who is at risk of care home admission is complex, relating to the current care and support of the person living with dementia as well as the availability and accessibility of care homes. Identifying evidence for prognostic factors for cognitive decline is also challenging given the wide variety in methodology in studies. This review and feedback from carers of people living with dementia and other stakeholders highlight that the risk of care home admission and cognitive decline is likely to be multifactorial. There are factors highlighted as of relevance by our stakeholder groups which have not been investigated sufficiently, or at all, and future research should assess these potential prognostic factors. We did identify potential inequalities in access to care home admission based on socio-demographic characteristics such as deprivation, race, and rurality, and several prognostic factors measurable in primary care that could alert clinicians that a person diagnosed with dementia may be at risk of a faster progression, with neuropsychiatric symptoms in particular being predictors of poorer prognosis.

## Supplementary Information

Supplementary Material 1.

## Data Availability

The datasets generated during the current study are available from the corresponding author on reasonable request.

## Abbreviations

AD: Alzheimer’s disease
ACE: Addenbrooke’s Cognitive Examination
BMI: Body Mass Index
CAMCOG: Cambridge Cognitive Examination
CD: Cognitive decline outcome
CHA: Care home admission outcome
CDR: Clinical Dementia Rating
CVD: Cardiovascular disease
DRS: Dementia Rating Scale
IQR: Inter Quartile Range
MEDDIP: Measurement of Dementia Disease Progression in Primary Care
MMSE: Mini Mental State Examination
3MS: Modified Mini Mental State examination
NSAIDs: Non-Steroidal Anti-Inflammatory Drugs
PPIE: Patient and Public Involvement and Engagement
PRISMA: Preferred reporting items for systematic reviews and meta-analyses
QUIPS: Quality in Prognostic Studies
RUG: Research User Group
SD: Standard deviation
