# Supplementary material for: Systematic review of prognostic factors for poor outcome in people living with dementia that can be determined from primary care medical records
Source: BMC Geriatr. 2024 Oct 1;24:801. doi: 10.1186/s12877-024-05389-0 (PMC11443862; doi:10.1186/s12877-024-05389-0)
Supplement: Supplementary file 1 — Supplementary Material 1. [file 12877_2024_5389_MOESM1_ESM.docx]

**Supplementary Materials**

## Supplementary Table 1. The search strategy used for Ovid Medline

| **Search terms** |
| --- |
| 1 Dementia/  2 Alzheimer Disease/  3 exp Dementia; Vascular/  4 exp frontotemporal dementia/  5 Lewy Body Disease/  6 dementia.ti;ab;kf.  7 alzheimer*.ti;ab;kf.  8 1 or 2 or 3 or 4 or 5 or 6 or 7  9 (cogniti* adj2 (declin* or reduc* or poor* or lower* or worse* or less*)).ti;ab;kf.  10 (neurological* adj2 (declin* or reduc* or poor* or lower* or less* or worse*)).ti;ab;kf.  11 (("Montreal Cognitive Assessment" or MoCA or "Mini-Mental State Exam*" or "Mini Mental State exam*" or MMSE or "Mini-Cog" or "Mini Cog" or "Addenbrooke* Cognitive Exam*" or "Mini ACE" or "Mini-ACE" or "Mini-Addenbrooke* Cognitive Exam*") adj2 (declin* or reduc* or poor* or lower* or worse* or less*)).ti;ab;kf.  12 9 or 10 or 11  13 (care adj1 (nursing or longer or long-term or "long term" or residen* or respite or home or homes or housing or facility or facilities or institution*)).ti;ab;kf.  14 (nursing adj1 (longer or long-term or "long term" or residen* or respite or home or homes or housing or facility or facilities or institution*)).ti;ab;kf.  15 ((longer or long-term or "long term") adj1 (residen* or respite or home or homes or housing or facility or facilities or institution*)).ti;ab;kf.  16 (residen* adj1 (respite or home or homes or housing or facility or facilities or institution*)).ti;ab;kf.  17 (respite adj1 (home or homes or housing or facility or facilities or institution*)).ti;ab;kf.  18 ((home or homes or housing) adj1 (facility or facilities or institution*)).ti;ab;kf.  19 exp Nursing Homes/  20 residential facilities/  21 Assisted Living Facilities/  22 homes for the aged/  23 Housing for the Elderly/  24 Respite Care/  25 Long-Term Care/  26 13 or 14 or 15 or 16 or 17 or 18 or 19 or 20 or 21 or 22 or 23 or 24 or 25  27 (admit* or admission or enter* or move or moves or moved or moving or "go* into" or "go* to" or "go* in" or transition or transfer*).ti;ab;kf.  28 26 adj3 27  29 Institutionalization/  30 28 or 29  31 Palliative Care/  32 Palliative Medicine/  33 Terminal Care/  34 Hospice care/  35 palliative.ti;ab;kf.  36 (supportive adj2 care).ti;ab;kf.  37 hospice*.ti;ab;kf.  38 31 or 32 or 33 or 34 or 35 or 36 or 37  39 Mortality/  40 Survival/  41 survival rate/  42 (death* or dying or died).ti;ab;kf.  43 mortality.ti;ab;kf.  44 survival.ti;ab;kf.  45 39 or 40 or 41 or 42 or 43 or 44  46 Prognosis/  47 prognos*.ti;ab;kf.  48 predict*.ti;ab;kf.  49 exp Cohort Studies/  50 cohort*.ti;ab;kf.  51 longitudinal.ti;ab;kf.  52 prospective.ti;ab;kf.  53 ("follow up" or follow-up).ti;ab;kf.  54 Disease Progression/  55 progression.ti;ab;kf.  56 ("disease course" or "natural course" or "natural history").ti;ab;kf.  57 "time series".ti;ab;kf.  58 46 or 47 or 48 or 49 or 50 or 51 or 52 or 53 or 54 or 55 or 56 or 57  59 12 or 30 or 38 [combined outcomes: cognitive decline or care home or palliative care]  60 8 and 58 and 59 [outcomes above + dementia + prognosis]  61 8 and 45 and 58 [mortality + dementia + prognosis]  62 limit 61 to dt=20180831-20220531 [limits mortality search from 31st Aug 2018 to 31st May 2022]  63 60 or 62 [both sets combined] |

## Supplementary Table 2. Strength of Evidence criteria adapted from GRADE

| **Strength of Evidence criteria adapted from GRADE** |
| --- |
| The start point for all assessments is that there is a **High** strength of evidence and downgrading to **Moderate**; **Low** or **Very Low** is made according to the following criteria:  **Risk of Bias**  Downgrade by 1 category if any of the studies included for the prognostic factor group has a QUIPs domain summary judged as having a high risk of bias.  **Inconsistency**  Downgrade by 1 category if any of the included studies have either:  1) Variations in the effect estimates for the prognostic factor with results on either side of the line of no effect  2) Heterogeneity between studies in the measures used for the prognostic factor or the outcomes.  **Indirectness**  Downgrade by 1 category if any of the included studies have any of:  1) Only a subset of the eligible population is represented (e.g., males or females)  2) The prognostic factor is not fully represented (e.g., only includes a subset)  3) The outcome is not fully measured for the whole target population (e.g., only a subset of those with cognitive decline or care home admissions are included.  **Imprecision**  Downgrade by 1 category if:  1) Only 1 study is included for the prognostic factor group  2) If analysis takes place in data that is from the same cohort  3) The prognostic factor is not included in all of the analysis (e.g., in the multivariable analysis). |

**Supplementary Table 3. Summary of study characteristics**

| **Paper ID** | **Cohort/study name; Study design** | **Country study conducted** | **Participants** | **Total number of participants; % female, mean age years** | **Mean age (years)** | **Living in institution** | **Length of follow-up** | **Outcomes measured** | **Prognostic factors measured** |
| --- | --- | --- | --- | --- | --- | --- | --- | --- | --- |
| Aguero-Torres 1998 | Kungsholmen; Prospective Cohort Study | Sweden | Aged ≥75  Dementia: 100% (AD & VD) | n=74  Female: 81%^a^ | 86.0 (SD 5.6)^a^ | 50%^a^ | 3 years | Cognitive Decline | Age; Sex; Education; Type of dementia; Cognition at baseline; Activities of daily living; Comorbidity. |
| Andel 2007 | Florida; Medical Record Data | USA | Aged ≥65 years  Dementia: 100% | n=587  Female: 74% | 78.9 (SD 8.0) | 0% | 4 years | Care Home Admission | Age; Sex; Race; Marital status; Self-reported general health;  Activities of daily living; Diabetes; Cerebrovascular disease (Stroke/TIA); Cardiovascular disease; Musculoskeletal disease; Cancer; Respiratory disease (Emphysema/COPD); Urinary incontinence; Currently receives care. |
| Bleckwenn 2017 | Ageing, cognition, and dementia in primary care patients (AgeCoDe) Study; Prospective Cohort Study | Germany | Age ≥75 years, Dementia: 100% (all AD) | n=113  Female: 75% | 85.57 (SD 3.3) | 0% | 3 years | Cognitive Decline | Age; Sex; Education; Cardiovascular Disease. |
| Charbonneau 2008 | Regie de l'Assurance Maladie du Quebec (RAMQ) database; Medical Record Data | Canada | Aged ≥66 years  Dementia: 100% (all AD) | n=17940  Female: 68% | Without CVD: 79.4 (SD 6.2);  With CVD: 80.3 (SD 6.0) | 0% | 1000 days | Care Home Admission | Cerebrovascular disease (stroke/TIA). |
| Contador 2017 | Neurological Disorders in Central Spain (NEDICES); Prospective Cohort Study | Spain | Aged ≥65 years  Dementia: 100% | N=53  Female: not reported | Not reported | 0% | 3 years | Cognitive Decline | Education. |
| Defrancesco 2020 | Prospective Dementia Registry Austria (PRODEM-Austria); Prospective Cohort Study | Austria | Dementia: 100% (all AD) | N=282  Female: 59.9% | 75.6 (SD 7.9) | 0% | 2 years | Cognitive Decline | Age; Sex; Sedative medication; Antidepressant medication; Antipsychotic medication; Depression/anxiety/irritability (emotional & affective factors); No of behavioural symptoms (elation, euphoria, apathy, disinhibition, aberrant motor behaviour); Psychosis. |
| Gibbons 2002 | Alzheimer's Disease Patient Registry (ADPR); Prospective Cohort Study | USA | Dementia: 100% (all AD) | N=372  Female: 64.0% | 78.6 (SD 6.4) | 0% | Mean 4.4 years (range 1-12 years) | Care Home Admission | Cognition; Inactivity; Psychosis (delusions/hallucinations/ suspiciousness/paranoia); Agitation/aggression (restlessness); Irritable/easy to anger; Aggressive behaviour; Depression; Anxiety/phobias; Fearful/apprehension; Tearful/cries; Social withdrawal. |
| Giebel 2021 | Secure Anonymised Information Linkage (SAIL) Demetia e-Cohort (SDEC); Retrospective Cohort Study | UK | Dementia: 100%  Admitted to care home in 2000-2018. | N=34514  Female: 68.3% | 87.0 (SD 7.0) | 100% (at least once during the study) | 1 year | Care Home Admission | Age at care home admission; Urban/rural residence; Deprivation; Living situation (alone, family); Frailty. |
| Guo 1999 | Kungsholmen Project; Prospective Cohort Study | Sweden | Aged ≥75 years  Dementia: 100% | N=79  Female: 78.4% | 82.5 (range 75-101) | 0% | 3 years (average) | Cognitive Decline | Diuretics. |
| Haaksma 2019 | Kungsholmen Project (KP) and Swedish National Study of Aging and Care in Kungsholmen (SNAC-K); Prospective Cohort Study | Sweden | Aged ≥75 years in KP cohort or ≥60 years in SNAC-K cohort  Dementia: 100% | N=509  Female: 78.3% | 88.3 (SD 5.3) | Not reported | Median 2.8 years (IQR 1.1-5.2) | Cognitive Decline | Age; Sex; Education; Type of Dementia; Comorbidity; Anticholinergic medication (burden); Social network. |
| Harsanyiova 2018 | (Bratislava Study); Prospective Cohort Study | Slovakia | Dementia: 100%  (VD or AD) | N=428  Female: 63.0% | 77.4 (SD 4.5) | 45% lived <30 days in nursing homes before 1st examination | 1 year | Cognitive Decline | Age; Sex; Living situation (alone, family, care home); Type of dementia. |
| Hebert 2001 | Canadian Study of Health and Aging (CSHA); Prospective Cohort Study | Canada | Aged ≥65 years  Dementia: 100% | N=293  Female: Not reported | Not reported | 0% | 5 years | Care Home Admission | Type of dementia; Cognition at baseline; Activities of daily living; Safety concerns; Urinary incontinence; No. of behaviour problems/psychiatric symptoms; Geographical region; Caregiver desire for admission (requests institutionalization); Caregiver age; Caregiver relationship; Caregiver burden; Caregiver health; Caregiver depressed; Caregiver living arrangement. |
| Helmes 2017 | Canadian Study of Health and Aging (CSHA); Prospective Cohort Study | Canada | Aged ≥65 year  Dementia: 100% (AD, VD or other type) | N=2133 at baseline  Female: Not reported | Not reported | Not reported | 5 and 10 years | Cognitive Decline | Age; Sex; Education; Urban/rural residence. |
| Helzner 2009 | Washington Heights Inwood Columbia Aging Project; Prospective Cohort Study | USA | Dementia: 100% (all AD) | N=156  Female: 71.2% | 83.0 (SD 6.4) | Not reported | Mean 3.5 years (SD 2.2, range 1.0-10.3) | Cognitive Decline | Smoking history; Cholesterol; Diabetes; Hypertension; Cerebrovascular disease (stroke/TIA); Cardiovascular disease. |
| Hope 1998 | (Oxfordshire Study); Prospective Cohort Study | UK | Dementia: 100%  (AD and/or VD) | N=50  Female: Not reported | Not reported | 0% | 1 year | Care Home Admission | Falls/gait problems (tendency to fall); Painkillers; Urinary incontinence; Bowel incontinence; Difficulty finding way about home; Night-time activity; Caregiver sex. |
| Jamieson 2020 | Home care International Residential Assessment Instrument (inter RAI-HC) assessment data; Prospective Cohort Study | New Zealand | Aged ≥65 years  Dementia: 100% | N=18672  Female: 56.2% | 81.1 (SD 6.9) | 0% | 5 years | Care Home Admission | Age; Sex; Marital status; Living situation (alone, family); Self-reported general health; Smoking (history of); Alcohol; Cognition at baseline; Activities of daily living; Falls; Gait speed; Diabetes; Cerebrovascular disease (stroke/TIA); Cardiovascular disease; Cancer; Respiratory disease (emphysema/ COPD); Hospitalisations; Hearing loss/impairment; Vision impairment/uses glasses; Urinary Incontinence; Bowel incontinence; Dyspnoea; Fatigue; Depression, anxiety and irritability; Psychosis (delirium); Major stress; Wandering; Lonely/time alone; Caregiver desire for admission (requests institutionalization); Weight/BMI. |
| Janssen 2020 | Netherlands Institute for Health Services Research Primary Care Database (NIVEL-PCD); Medical Record Data | Netherlands | Adults born ≤1965 Dementia: 100% | N=11012  Female: 61.0% | 79.8 (SD 8.0) | 0% | Mean 1.9-3.1 years by age group | Care Home Admission | Age; Sex; Migrant status; Income; Living situation (alone, family); Dementia medication; No. of medications/polypharmacy. |
| Joling 2020 | Netherlands Institute for Health Services Research Primary Care Database (NIVEL-PCD); Medical Record Data | Netherlands | Adults born ≤1965 Dementia: 100% | N=9230  Female: 60.3% | 79.7 (SD 7.9) | 0% | Median 2.0 years (IQR 2.2) | Care Home Admission | Baseline age; Sex; Migrant status; Living situation (alone, family); Frailty; Taking dementia medication;  Polypharmacy (No. of medications); Currently receives care (after diagnosis); Care at home before diagnosis. |
| Kadlec 2018 | Alzheimer’s Drug Therapy Initiative (ATDI) and British Colombia Ministry of Health; Medical Record Data | Canada | Aged ≥50 years  Dementia: 100% | N=10475  Female: 60.2% | 80.2 (SD 7.2) | 0% | 2.5 years | Cognitive Decline    Care Home Admission | Education; Cognition at baseline. |
| Leoutsakos 2012 | Cache County Dementia Progression Study; Prospective Cohort Study | USA | Aged ≥65 years  Dementia: 100%  (all AD) | N=335  Female: 34% | 85.96 (SD 6.3) | Not reported | Median 3.1 years (range 0.7, 11.2) | Cognitive Decline | Age; Sex; Education; Self-reported general health; Duration of dementia; Polypharmacy (No. of medications); Comorbidity. |
| Leoutsakos 2015 | Cache County Dementia Progression Study; Prospective Cohort Study | USA | Aged ≥65 years  Dementia: 100%  (all AD) | N=328  Female: 66% | 84.3 (SD 6.4) | Not reported | Mean 3.7 years (SD 2.5) | Cognitive Decline | Age at onset; Sex; Education; Duration of dementia; Cognition at baseline; Functional activity; Hypertension; Cerebrovascular disease (stroke/TIA); Psychosis; Depression; Apathy. |
| Luppa 2012 | The German Study on Ageing, Cognition and Dementia in Primary Care Patients (AgeCoDe); Prospective Cohort Study | Germany | Aged ≥75 years  Dementia: 100% | N=254  Female: 67.0% | Institutionalised: 84.6 (SD 4.6)  Non-institutionalised: 83.5 (SD 3.8) | 0% | Up to 6 years  (Follow up on average 4 times every 1.5 years) | Care Home Admission | Age; Sex; Marital status; Education; Cognition at baseline; Mobility; Hearing loss/impairment; Vision impairment/uses glasses. |
| MacDonald 2011 | Kungsholmen Project; Prospective Cohort Study | Sweden | Aged ≥75 years  Dementia: 100% | N=308  Female: Not reported | 87.6 (age at onset) | Not reported | Up to 3 follow-ups at 3-year intervals (max. 9 years) | Cognitive Decline | Age at onset; Sex; Education; Frailty; Weight/BMI; Cognition at baseline; Family history of dementia; Comorbidity; Diabetes; Hypertension; Cardiovascular disease; NSAIDs; Antihypertensive medication; Hearing loss/impairment; Depression; Social network. |
| Melis 2013 | Kungsholmen Project ; Prospective Cohort Study | Sweden | Aged ≥75 years  Dementia: 100% | N=310  Female: 83.2% | 85.0 (SD 4.5) (in dementia cohort) | 3.90% | Mean 4.4 years (SD 2.0)  (max. 9 years and  4 follow-ups) | Cognitive Decline | Comorbidity. |
| Mortimer 1992 | Minneapolis Study; Prospective Cohort Study | USA | Dementia: 100%  (all AD) | N=65  Female: 22.0% | 63.8 (SD 8.5) (age at onset) | 0% | 3 years | Cognitive Decline | Age; Sex; Education; Cognition at baseline; Hypertension or stroke; Extrapyramidal signs; Depression/anxiety/irritability (affective disorders); Psychosis (hallucination/paranoia); Aggressive/agitation; Sleep disorders/disturbance; Activity disturbance. |
| Nerius 2018 | Allgemeine Orstrankenkassse (AOK); Medical Record Data | Germany | Aged ≥60 years  Dementia: 100% | N=9950  Female: 65.0% | 80.1 (SD 7.7) | 0% | Mean 18.4 months | Care Home Admission | Age; Sex; Dementia medication; Cholesterol; Diabetes; Hypertension; Cerebrovascular disease (stroke/TIA); Cardiovascular disease; Cancer; Polypharmacy (No. of medications); Antipsychotic medication; Currently receives care. |
| Nygaard 1991 | Bergen Study; Prospective Cohort Study | Norway | Dementia: 100% | N=46  Female: 71.7% | 81.1 (SD 6.6) | 0% | 1 year | Care Home Admission | Age; Sex; Marital status; Living situation (alone; family); Duration of dementia; Cognition at baseline; Difficulty understanding; Difficulties communicating; Depression; Safety concerns; Eating problems; Urinary incontinence; Bowel incontinence; Mental health (composite); Psychosis (delusions/ hallucinations/suspiciousness/ paranoia); Agitation (restlessness); Aggression (hitting); Sleep disturbance/disorders; Apathy/listless; Wandering (and purposeless and inappropriate activities); Inappropriate urination; Difficulty finding way about home; Currently receives care; Amount of care/help required; Caregiver desire for admission (request institutionalization); Caregiver sex; Caregiver age; Caregiver relationship; Caregiver burden; Caregiver main income; Caregiver physical health; Caregiver mental health (despair/anger/ depression/anxiety); Caregiver sleep disturbances; Caregiver loss of hobbies; Caregiver guilt; Caregiver not enough time for self; Caregiver family conflict; Caregiver difficulty taking responsibility; Caregiver employed. |
| Peters 2015 | Cache County Study; Prospective Cohort Study | USA | Aged ≥65 years  Dementia: 100% | N=335  Female: Not reported | 84.3 (SD 6.4) | Not reported | 16 years | Cognitive Decline | Age at onset; Sex; Education; Self-reported general health; Time from onset of symptoms to diagnosis; Aggression/agitation; Depression/anxiety/irritability; Psychosis; Apathy; Severe neuropsychiatric symptoms. |
| Rathod-Mistry 2021 | MEDDIP; Retrospective Cohort Study | UK | Dementia: 100%  (AD, VD, Lewy body, Parkinson’s, frontotemporal; mixed; unspecified) | N=30463  Female: 63.5% | 81.6 (SD 7.9) | 0% | 21 years | Palliative Care | Diet/nutrition; Cognition; Dementia medication; Functional activity; Safety concerns; Comorbidity; No. of medications/ polypharmacy; No. of symptoms; Mental health (composite); Caregiver/receiving care; Home pressures; Severe neuropsychiatric; Imaging. |
| Razay 2009 | Oxford Project to Investigate Memory and Aging (OPTIMA); Prospective Cohort Study | UK | Aged ≥60 years  Dementia: 100% (AD, other dementia type) | N=200  Female: 50% AD; 44% other dementia | AD: 74.1 (SD 6.7)    Other dementia: 74.2 (SD 7.9) | Not reported | Up to 5 years | Cognitive Decline | Antihypertensive. |
| Read 2021 | ELSA; Prospective Cohort Study | UK | Aged ≥50 years  Dementia: 100% | N=234  Female: 52.0% | 78.4 (SD 9.5) | 11% | 4 years | Care Home Admission | Activities of daily living; Activities of daily living (unmet need). |
| Ruitenberg 2001 | Rotterdam Study; Prospective Cohort Study | Netherlands | Aged ≥55 years  Dementia: 100% | N=401 (147 for care home)  Female: 77.1% | 84.9 (SD 6.5) | 69% | Mean 2.2 years (SD 1.3) | Cognitive Decline    Care Home Admission | Age; Sex; Education (Cognitive outcome only); Cognition (care home only). |
| Runte 2018 | BARMER GEK; Medical Record Data | Germany | Aged ≥60 years  Dementia: 100% | N=652  Female: 51.1% | 81.2 (SD 7) | 0% | Mean 2.2 years (SD 1.4)  Max. 4.5 years | Care Home Admission | Age; Sex; Self-reported general health; Cognition; Activities of daily living; Comorbidity; Mental health (composite); Currently receives care; Caregiver sex; Caregiver age; Caregiver relationship; Caregiver living arrangement; Support available; Quality (Impact) of support. |
| Scarmeas 2006 | New York Study; Prospective Cohort Study | USA | Aged >65 years  Dementia: 100% | N=312  Female: 73.0% | 81.6 (SD 6.8) | 0% | 13 years | Cognitive Decline | Age; Sex; Race; Education; Literacy; Smoking (history of); Cognition; Cholesterol; Diabetes; Hypertension; Cerebrovascular disease (stroke/TIA); Cardiovascular disease. |
| Schmidt 2013 | Rapidly Progressing Alzheimer's Disease study (rpAD); Prospective Cohort Study | Germany | Dementia: 100% (all AD) | N=78  Female: 60.0% | 65.0 (SD 10.0) | Not reported | 1 year | Cognitive Decline | Pre-progression rate |
| Severson 1994 | Alzheimer's Disease Patient Registry (ADPR); Prospective Cohort Study | USA | Aged ≥60 years  Dementia: 100% | N=136  Female: 67.0% | 79.9 (SD 8.4) | 0% (those in a nursing home excluded from analysis) | Mean 1.5 years (1^st^ follow up)    Mean 3.9 years (2^nd^ follow up) | Care Home Admission | Age; Sex; Marital status; Cognition; Activities of daily living; Size of support network. |
| Small 1997 | Kungsholmen Project; Prospective Cohort Study | Sweden | Aged ≥75 years  Dementia: 100% (AD, VD only) | N=36  Female: 77.8% | 83.0 (SD 5.20) | Not reported | 2.5 years (approx.) | Cognitive Decline | Age; Sex; Education; Type of dementia; Cognition at baseline (IQ). |
| Smith 2000 | Mayo Clinic Medical Record Study; Retrospective Cohort Study | USA | Aged >65 years  Dementia: 100% | N=220 (323 controls  Female: 64.6% | 80.8 (SD 7.1) | 27% | 11 years | Care Home Admission | Age; Sex; Marital status; Type of residence; Education; Comorbidity; Living situation; Hospitalisation; No. of doctor visits. |
| Suh 2004 | Dementia Patient Registry; Prospective Cohort Study | Korea | Aged >50 years  Dementia: 100% (all AD) | N=107  Female: 78.5% | 79.5 (SD 9.8) | Not reported | 1 year | Cognitive Decline | Age; Sex; Education; Duration since onset; Cognition; Comorbidity (other health problems). |
| Taylor 2017 | Sydney Study; Prospective Cohort Study | Australia | Aged >60 years  Dementia: 100% | N=115  Female: 59.0%^a^ | 82.2^a^ (SD 6.7) | Not reported | 1 year | Cognitive Decline | Gait speed |
| Teri 1995 | Alzheimer’s Disease Patient Registry; Medical Record Data | USA | Dementia: 100% (all AD) | N=156  Female: 67.0% | 79 (range 54-91) | Not reported | Average 3 years | Cognitive Decline | Age; Sex; Education; Duration of dementia; Cognition; Cognition over follow-up; Falls/gait problems (tendency to fall); Inactivity; Comorbidity; Cardiovascular disease (peripheral vascular); Musculoskeletal disease; Vision impairment (uses glasses); No. of behaviour problems; Psychosis (paranoia); Agitation/restlessness/loud; Irritable/easy to anger; Complaining/ Critical/Demanding; Depression; Fearful/apprehension; Tearful/ cries; Problems sleeping; Emotionally labile; Paces; Wandering (gets lost); Hoards things; Social withdrawal; Difficulty with finances/handling money; Difficulty dressing. |
| Treiber 2011 | The Cache County Dementia Progression Study; Prospective Cohort Study | USA | Aged ≥65 years  Dementia: 100% | N=187  Female: 64.7% | 84.6 (SD 5.8) | 0% | Mean 2.7 years (SD 0.4)) | Cognitive Decline | Education; Cognition at baseline (IQ); Occupation; Duration of dementia; No. of cognitive activities. |
| Tschanz 2011 | Cache County Study on Memory in Aging (CCSMA) Dementia Progression Study (DPS); Prospective Cohort Study | USA | Aged ≥65 years  Dementia: 100%  (all AD) | N=328  Female: 66.0% | 85.9 (SD 6.3) | 19% | Mean 3.8 years (range 0.07, 12.9) | Cognitive Decline | Age at onset; Sex; Education. |
| Wetmore 2021 | Medicare Study; Medical Record Data | USA | Aged ≥40 years  Dementia: 100% | N=154380 (Matched 1:4 psychosis: non-psychosis patients)  Female: 70.3% | 82.5 (SD 7.2) | 0% | Mean 1.6 years (SD 1.6) for psychosis patients  Mean 2.2 years (SD 1.9) for non-psychosis patients  Max 9 years | Care Home Admission | Age; Sex; Race; Mobility (Hemiplegia/paraplegia); Diabetes; Cerebrovascular disease (stroke/TIA); Cardiovascular disease; Musculoskeletal (rheumatological) disease; Cancer; Respiratory disease (emphysema/ COPD); Peptic ulcer disease; Liver disease; Renal disease; Psychosis. |
| Williams 2006 | St Louis Study; Prospective Cohort Study | USA | Dementia: 100%  (AD, Lewy body) | N=315  Female: 58.0% | 73.8 (age at onset)  AD: 74.6 (SD 10.3)  DLB: 70.8 (SD 9.4) | Not reported | Until death (intervals not reported) | Care Home Admission | Sex; Education; Type of dementia; Cognition; Activities of daily living; Falls/gait problems (tendency to fall); Comorbidity; Extrapyramidal signs; Depression; Polypharmacy (No. of medications); Weight. |
| Zafeiridi 2021 | Medical Record Data; Retrospective Cohort Study | Northern Ireland | Dementia: 100% | N=25418  Female: 65.1% | 77.3 (SD 8.3) | 0% | 7 years | Care Home Admission | Age; Sex; Marital status; Urban/rural residence; Deprivation; Dementia medication; Hospitalisation. |

## *^a^ based on whole cohort which will be larger than number given which refers to number in analysis, SD; Standard deviation; AD; Alzheimer’s disease; CVD; Cardiovascular disease; IQR; Inter Quartile Range.*

## Supplementary Table 4. References of papers included in the review

| **References** |
| --- |
| Aguero-Torres, H.; Fratiglioni, L.; Guo, Z. Prognostic factors in very old demented adults. (1998) J Am Geriatr Soc, 46(4):4442-52. |
| Andel, R.; Hyer, K.; Slack, A. Risk factors for nursing home placement in older adults with and without dementia. (2007) J Aging Health, 19(2):213-28. |
| Bleckwenn, M.; Kleineidam, L.; Wagner, M.; Jessen, F.; Weyerer, S.; Werle, J.; Wiese, B.; Luhmann, D.; Posselt, T.; Konig, H. H.; Brettschneider, C.; Mosch, E.; Weeg, D.; Fuchs, A.; Pentzek, M.; Luck, T.; Riedel-Heller, S. G.; Maier, W. Impact of coronary heart disease on cognitive decline in Alzheimer's disease: a prospective longitudinal cohort study in primary care. (2017) Br J Gen Pract, 67(655):e111-7. |
| Charbonneau, C.; Massoud, F.; Dorais, M.; LeLorier, J. A retrospective study of cholinesterase inhibitors for Alzheimer's disease: cerebrovascular disease as a predictor of patient outcomes. (2008) Curr Med Res Opin, 24(12):3287-94. |
| Contador, I.; Bermejo-Pareja, F.; Pablos, D. L.; Villarejo, A.; Benito-Leon, J. High education accelerates cognitive decline in dementia: A brief report from the population-based NEDICES cohort. (2017) Dement Neuropsychol, 11(3):297-300. |
| Defrancesco, M.; Marksteiner, J.; Kemmler, G.; Dal-Bianco, P.; Ransmayr, G.; Benke, T.; Mosbacher, J.; Holler, Y.; Schmidt, R. Specific Neuropsychiatric Symptoms Are Associated with Faster Progression in Alzheimer's Disease: Results of the Prospective Dementia Registry (PRODEM-Austria). (2020) J Alzheimers Dis, 73(1):125-33. |
| Gibbons, L. E.; Teri, L.; Logsdon, R.; McCurry, S. M.; Kukull, W.; Bowen, J. Anxiety symptoms as predictors of nursing home placement in patients with Alzheimer's disease. (2002) Journal of Clinical Geropsychology, 8(4):335-42. |
| Giebel, C.; Hollinghurst, J.; Akbari, A.; Schnier, C.; Wilkinson, T.; North, L.; Gabbay, M.; Rodgers, S. Socio-economic predictors of time to care home admission in people living with dementia in Wales: A routine data linkage study. (2021) Int J Geriatr Psychiat, 36(4):511-20. |
| Guo, Z.; Fratiglioni, L.; Zhu, L.; Fastbom, J.; Winblad, B.; Viitanen, M. Occurrence and progression of dementia in a community population aged 75 years and older: relationship of antihypertensive medication use. (1999) Arch Neurol, 56(8):991-6. |
| Haaksma, M. L.; Rizzuto, D.; Leoutsakos, J. M. S.; Marengoni, A.; Tan, E. C. K.; Olde Rikkert, M. G. M.; Fratiglioni, L.; Melis, R. J. F.; Calderon-Larranaga, A. Predicting Cognitive and Functional Trajectories in People With Late-Onset Dementia: 2 Population-Based Studies. (2019) J Am Med Dir Assoc, 20(11):1444-50. |
| Harsanyiova, M.; Prokop, P. Living condition, weight loss and cognitive decline among people with dementia. (2018) Nurs, 5(3):275-284. |
| Hebert, R.; Dubois, M.; Wolfson, C. Factors associated with long-term institutionalization of older people with dementia. (2001) Journals of Gerontology: Series A, Biological Sciences and Medical Sciences, 56(11):M693-9. |
| Helmes, E.; Van Gerven, P. W. M. Urban residence and higher education do not protect against cognitive decline in aging and dementia: 10-year follow-up of the Canadian Study of Health and Aging. (2017) Educational Gerontology, 43(11):552-60. |
| Helzner, E. P.; Luchsinger, J. A.; Scarmeas, N.; Cosentino, S.; Brickman, A. M.; Glymour, M. M.; Stern, Y. Contribution of vascular risk factors to the progression in Alzheimer disease. (2009) Arch Neurol, 66(3):343-8. |
| Hope, T.; Keene, J.; Gedling, K.; Fairburn, C. G.; Jacoby, R. Predictors of institutionalization for people with dementia living at home with a carer. (2018) Int J Geriatr Psychiat, 13(10):682-90. |
| Jamieson, H.; Abey-Nesbit, R.; Nishtala, P. S.; Allore, H.; Han, L.; Deely, J. M.; Pickering, J. W. Predictors of Residential Care Admission in Community-Dwelling Older People With Dementia. (2020) J Am Med Dir Assoc, 21(1):1665-70. |
| Janssen, O.; Vos, S. J. B.; Handels, R.; Vermunt, L.; Verheij, R.; Verhey, F. R. J.; van Hout, H.; Visser, P. J. Duration of Care Trajectories in Persons With Dementia Differs According to Demographic and Clinical. (2020) J Am Med Dir Assoc, 21(8):1102-7. |
| Joling, K. J.; Janssen, O.; Francke, A. L.; Verheij, R. A.; Lissenberg-Witte, B. I.; Visser, P. J.; van Hout, H. P. J. Time from diagnosis to institutionalization and death in people with dementia. (2020) Alzheimer's dement, 16(4):662-671. |
| Kadlec, H.; Dujela, C.; Beattie, B. L.; Chappell, N. Cognitive functioning, cognitive reserve, and residential care placement in patients with Alzheimer's and related dementias. (2018) Aging Ment Health, 22(1):19-25. |
| Leoutsakos, J. M. S.; Forrester, S. N.; Corcoran, C. D.; Norton, M. C.; Rabins, P. V.; Steinberg, M. I.; Tschanz, J. T.; Lyketsos, C. G. Latent classes of course in Alzheimer's disease and predictors: The Cache County Dementia Progression Study. (2015) Int J Geriatr Psychiat, 30(8):824-32. |
| Luppa, M.; Riedel-Heller, S. G.; Stein, J.; Leicht, H.; Konig, H. H.; van den Bussche, H.; Maier, W.; Scherer, M.; Bickel, H.; Mosch, E.; Werle, J.; Pentzek, M.; Fuchs, A.; Eisele, M.; Jessen, F.; Tebarth, F.; Wiese, B.; Weyerer, S. Predictors of institutionalisation in incident dementia--results of the German Study on Ageing, Cognition and Dementia in Primary Care Patients (AgeCoDe study). (2012) Dement Geriatr Cogn Disord, 33(4):282-8. |
| MacDonald, S. W.; Karlsson, S.; Fratiglioni, L.; Backman, L. Trajectories of cognitive decline following dementia onset: what accounts for variation in progression? (2011) Dement Geriatr Cogn Disord, 31(3):202-9. |
| Melis, R. J. F.; Marengoni, A.; Rizzuto, D.; Teerenstra, S.; Kivipelto, M.; Angleman, S. B.; Fratiglioni, L. The influence of multimorbidity on clinical progression of dementia in a population-based cohort. (2013) PLoS ONE, 8(12):e84014. |
| Mortimer, J. A.; Ebbitt, B.; Jun, S. P.; Finch, M. D. Predictors of cognitive and functional progression in patients with probable Alzheimer's disease. (1992) Neurology, 42(9):1689-96. |
| Nerius, M.; Johnell, K.; Garcia-Ptacek, S.; Eriksdotter, M.; Haenisch, B.; Doblhammer, G. The Impact of Antipsychotic Drugs on Long-term Care, Nursing Home Admission, and Death in Dementia Patients. (2018) J Gerontol A Biol Sci Med Sci, 73(10):1396-1402. |
| Ono, R.; Uchida, K.; Nakatsuka, K.; Megumi, M.; Fukuda, H. Economic Status and Mortality in Patients with Alzheimer's Disease in Japan: The Longevity Improvement and Fair Evidence Study. (2022) J Am Med Dir Assoc, 23(1): 161-4. |
| Peters, M. E.; Schwartz, S.; Han, D.; Rabins, P. V.; Steinberg, M.; Tschanz, J. T.; Lyketsos, C. G. Neuropsychiatric symptoms as predictors of progression to severe Alzheimer's dementia and death: the Cache County Dementia Progression Study. (2015) Am J Psychiat, 172(5):460-5. |
| Piovezan, R. D.; Oliveira, D.; Arias, N.; Acosta, D.; Prince, M. J.; Ferri, C. P. Mortality Rates and Mortality Risk Factors in Older Adults with Dementia from Low- and Middle-Income Countries: The 10/66 Dementia Research Group Population-Based Cohort Study. (2020) J Alzheimers Dis, 75(2):581-93. |
| Rathod-Mistry, T.; Marshall, M.; Campbell, P.; Bailey, J.; Chew-Graham, C. A.; Croft, P.; Frisher, M.; Hayward, R.; Negi, R.; Robinson, L.; Singh, S.; Sumathipala, A.; Thein, N.; Walters, K.; Weich, S.; Jordan, K. P. Indicators of dementia disease progression in primary care: An electronic health record cohort study. (2021) Eur J Neurol, 28(5):1499-1510. |
| Razay, G.; Williams, J.; King, E.; Smith, A. D.; Wilcock, G. Blood pressure, dementia and Alzheimer's disease: the OPTIMA longitudinal study. (2009) Dement Geriatr Cogn Disord, 28(1):70-4. |
| Read, S.; Hu, B.; Wittenberg, R.; Brimblecombe, N.; Robinson, L.; Banerjee, S. A Longitudinal Study of Functional Unmet Need Among People with Dementia. (2021) J Alzheimers Dis, 84(2):705-16. |
| Ruitenberg, A.; Kalmijn, S.; de Ridder, M. A.; Redekop, W. K.; van Harskamp, F.; Hofman, A.; Launer, L. J.; Breteler, M. M. Prognosis of Alzheimer's disease: the Rotterdam Study. (2001) Neuroepidemiology, 20(3):188-95. |
| Sarycheva, T.; Lavikainen, P.; Taipale, H.; Tiihonen, J.; Tanskanen, A.; Hartikainen, S.; Tolppanen, A. M. Antiepileptic drug use and mortality among community-dwelling persons with Alzheimer disease. (2020) Neurology, 94(20):e2099-2108. |
| Scarmeas, N.; Albert, S. M.; Manly, J. J.; Stern, Y. Education and rates of cognitive decline in incident Alzheimer's disease. (2006) J Neurol Neurosur Ps, 77(3):161-4. |
| Schmidt, C.; Karch, A.; Artjomova, S.; Hoeschel, M.; Zerr, I. Pre-progression rates in alzheimer's disease revisited. (2013) J Alzheimers Dis, 35(3):451-4. |
| Severson, M. A.; Smith, G. E.; Tangalos, E. G.; Petersen, R. C.; Kokmen, E.; Ivnik, R. J.; Atkinson, E. J.; Kurland, L. T. Patterns and predictors of institutionalization in community-based dementia patients. (1994) J Am Geriatr Soc, 1(2):181-5. |
| Small, B. J.; Viitanen, M.; Winblad, B.; Backman, L. Cognitive changes in very old persons with dementia: The influence of demographic, psychometric, and biological variables. (1997) J Clin Exp Neuropsyc, 19(2):245-60. |
| Smith, G. E.; Kokmen, E.; O'Brien, P. C. Risk factors for nursing home placement in a population-based dementia cohort. (2000) J Am Geriatr Soc, 48(5):519-25. |
| Suh, G. H.; Ju, Y. S.; Yeon, B. K.; Shah, A. A longitudinal study of Alzheimer's disease: Rates of cognitive and functional decline. (2004) Int J Geriatr Psychiat, 19 (9):817-24. |
| Taylor, M. E.; Lasschuit, D. A.; Lord, S. R.; Delbaere, K.; Kurrle, S. E.; Mikolaizak, A. S.; Kvelde, T.; Close, J. C. T. Slow gait speed is associated with executive function decline in older people with mild to moderate dementia: A one year longitudinal study. (2017) Arch Gerontol Geriatr, 73:148-53. |
| Teri, L.; McCurry, S. M.; Edland, S. D.; Kukull, W. A.; Larson, E. B. Cognitive decline in Alzheimer's disease: a longitudinal investigation of risk factors for accelerated decline. (1995) Journals of Gerontology Series A: Biological Sciences & Medical Sciences, 50(1):M49-55. |
| Treiber, K. A.; Carlson, M. C.; Corcoran, C.; Norton, M. C.; Breitner, J. C. S.; Piercy, K. W.; DeBerard, M. S.; Stein, D.; Foley, B.; Welsh-Bohmer, K. A.; Frye, A.; Lyketsos, C. G.; Tschanz, J. T. Cognitive Stimulation and Cognitive and Functional Decline in Alzheimer's Disease: The Cache County Dementia Progression Study. (2011) Journals of Gerontology: Series B: Psychological Sciences and Social Sciences, 66(4):416-25. |
| Tschanz, J. T.; Corcoran, C. D.; Schwartz, S.; Treiber, K.; Green, R. C.; Norton, M. C.; Mielke, M. M.; Piercy, K.; Steinberg, M.; Rabins, P. V.; Leoutsakos, J.; Welsh-Bohmer, K. A.; Breitner, J. C. S.; Lyketsos, C. G. Progression of Cognitive, Functional, and Neuropsychiatric Symptom Domains in a Population Cohort With Alzheimer Dementia: The Cache County Dementia Progression Study. (2011) Am J Geriatr Psychiatry, 19(6):532-42. |
| Wetmore, J. B.; Peng, Y.; Yan, H.; Li, S.; Irfan, M.; Shim, A.; Roetker, N. S.; Abler, V.; Rashid, N. Association of Dementia-Related Psychosis With Long-term Care Use and Death. (2021) Neurology, 96(12):e1620-31. |
| Williams, M. M.; Xiong, C.; Morris, J. C.; Galvin, J. E. Survival and mortality differences between dementia with Lewy bodies vs Alzheimer disease. (2006) Neurology, 67(11):1935-41. |
| Zafeiridi, E.; McMichael, A. J.; Passmore, A. P.; McGuinness, B. Factors influencing transition to care homes for people with dementia in Northern Ireland. (2021) Alzheimers Dement (N Y), 7(1):e12120. |

## Supplementary Table 5. Risk of Bias (QUIPS): Domain summary assessments

| **Study** | **Participation Domain** | **Attrition Domain** | **Prognostic Factor Domain** | **Outcome Domain** | **Confounding Domain** | **Statistical Analysis Domain** | **Overall**  **Risk of Bias** |
| --- | --- | --- | --- | --- | --- | --- | --- |
| Aguero-Torres 2000 | **Low** | **High** | **Low** | **Low** | **Low** | **Low** | **High** |
| Andel 2007 | **Low** | **Low** | **Low** | **Low** | **Low** | **Low** | **Low** |
| Bleckwenn 2018 | **Low** | **Moderate** | **Low** | **Low** | **Low** | **Low** | **Moderate** |
| Charbonneau 2008 | **Low** | **Moderate** | **Low** | **Moderate** | **Moderate** | **Moderate** | **Moderate** |
| Contador 2018 | **Moderate** | **Moderate** | **Low** | **Low** | **High** | **Moderate** | **High** |
| Defrancesco 2021 | **Moderate** | **High** | **Moderate** | **Low** | **High** | **Low** | **High** |
| Gibbons 2002 | **Moderate** | **Moderate** | **Moderate** | **Moderate** | **Low** | **Low** | **Moderate** |
| Giebel 2022 | **Low** | **Moderate** | **Low** | **Low** | **Moderate** | **High** | **High** |
| Guo 2000 | **Moderate** | **High** | **Low** | **Low** | **Low** | **Moderate** | **High** |
| Haaksma 2019 | **Low** | **High** | **Low** | **Moderate** | **Low** | **Low** | **High** |
| Harsanyiova 2019 | **Moderate** | **High** | **Low** | **Low** | **Low** | **Moderate** | **High** |
| Hebert 2002 | **Moderate** | **Moderate** | **Low** | **Low** | **Moderate** | **Low** | **Moderate** |
| Helmes 2017 | **Moderate** | **High** | **Moderate** | **Low** | **Moderate** | **Low** | **High** |
| Helzner 2009 | **Moderate** | **Moderate** | **Low** | **Low** | **Low** | **Low** | **Moderate** |
| Hope 1999 | **Moderate** | **High** | **Moderate** | **Low** | **High** | **High** | **High** |
| Jamieson 2021 | **Moderate** | **Moderate** | **Moderate** | **Low** | **Low** | **Low** | **Moderate** |
| Janssen 2020 | **Low** | **Low** | **Low** | **Low** | **Moderate** | **Low** | **Moderate** |
| Joling 2020 | **Low** | **Moderate** | **Low** | **Low** | **Low** | **Low** | **Moderate** |
| Kadlec 2019 | **Moderate** | **Moderate** | **Low** | **Low** | **High** | **Moderate** | **High** |
| Leoutsakos 2012 | **Moderate** | **High** | **Low** | **Low** | **Low** | **Low** | **High** |
| Leoutsakos 2015 | **Moderate** | **High** | **Low** | **Low** | **Moderate** | **High** | **High** |
| Luppa 2012 | **Low** | **High** | **Moderate** | **Low** | **Moderate** | **Low** | **High** |
| MacDonald 2011 | **Moderate** | **High** | **High** | **Moderate** | **Moderate** | **Low** | **High** |
| Melis 2013 | **Moderate** | **Moderate** | **Moderate** | **Low** | **Moderate** | **Low** | **Moderate** |
| Mortimer 1993 | **Low** | **Moderate** | **Low** | **Low** | **Low** | **Low** | **Moderate** |
| Nerius 2018 | **Low** | **Low** | **Low** | **Moderate** | **Moderate** | **Low** | **Moderate** |
| Nygaard 1991 | **Moderate** | **Moderate** | **High** | **Moderate** | **High** | **High** | **High** |
| Peters 2016 | **Moderate** | **Moderate** | **Low** | **Low** | **Low** | **Low** | **Moderate** |
| Rathod-Mistry 2022 | **Low** | **Low** | **Low** | **Low** | **Moderate** | **Low** | **Moderate** |
| Razay 2009 | **High** | **High** | **Moderate** | **Low** | **Low** | **Low** | **High** |
| Read 2022 | **Low** | **Low** | **Low** | **Low** | **Low** | **Low** | **Low** |
| Ruitenberg 2002 | **Low** | **Moderate** | **Low** | **Low** | **Low** | **Low** | **Moderate** |
| Runte 2018 | **Moderate** | **Low** | **Low** | **Moderate** | **Low** | **Low** | **Moderate** |
| Scarmeas 2007 | **Moderate** | **High** | **Low** | **Low** | **Low** | **Moderate** | **High** |
| Schmidt 2014 | **High** | **Moderate** | **Low** | **Low** | **Low** | **Low** | **High** |
| Severson 1994 | **Moderate** | **High** | **Low** | **Low** | **Low** | **Moderate** | **High** |
| Small 1997 | **Low** | **Moderate** | **Moderate** | **Low** | **Moderate** | **Low** | **Moderate** |
| Smith 2000 | **Low** | **Moderate** | **Low** | **Low** | **Moderate** | **Moderate** | **Moderate** |
| Suh 2005 | **Moderate** | **High** | **Low** | **Low** | **Low** | **Low** | **High** |
| Taylor 2017 | **Moderate** | **High** | **Low** | **Low** | **Moderate** | **Low** | **High** |
| Teri 1995 | **Moderate** | **High** | **High** | **Low** | **Moderate** | **Moderate** | **High** |
| Treiber 2012 | **Low** | **High** | **Low** | **Low** | **Low** | **Moderate** | **High** |
| Tschanz 2012 | **Low** | **High** | **Low** | **Low** | **Moderate** | **Moderate** | **High** |
| Wetmore 2021 | **Moderate** | **Moderate** | **Low** | **Low** | **Moderate** | **Moderate** | **Moderate** |
| Williams 2006 | **Moderate** | **Moderate** | **Moderate** | **Moderate** | **High** | **Moderate** | **High** |
| Zafeiridi 2022 | **Low** | **Low** | **Moderate** | **Low** | **Low** | **Moderate** | **Moderate** |

# Supplementary Table 6. GRADE assessments: summary of results for care home admission

Total number of studies = 21; 94 factors, total sample size = 294,896

|  | **Prognostic Factor** | **Number of studies^a^ and associations^b^** | **Factor associations** | **Risk of Bias (RoB) summary** | **Consistency** | **Directness** | **Precision** | **Final GRADE** | **Factor summary** |
| --- | --- | --- | --- | --- | --- | --- | --- | --- | --- |
| **Sociodemographic factors** | **Age** | 14 studies;  31 associations | 19 significant associations; Older age higher risk | No concern: High RoB in 4/14 studies | Downgrade: Variation in measures | No concern | No concern | **Moderate** | Moderate evidence that older age increases risk of care home admission |
|  | **Sex** | 14 studies;  16 associations | 6 significant associations; Females sex higher risk | No concern: High RoB in 4/14 studies | No concern | No concern | No concern | **High** | High evidence but inconsistent associations between sex and risk of care home admission |
|  | **Education** | 4 studies;  6 associations | 3 significant associations; More years 2 studies higher risk, 1 study lower risk | Downgrade: High RoB in 3/4 studies | Downgrade: Results inconsistent | No concern | No concern | **Low** | Low evidence but Inconsistent associations between education and the risk of care home admission |
|  | **Marital status** | 7 studies;  17 associations | 6 significant associations; 2 married higher risk, 2 widowed/divorced/never married higher risk, 2 single higher risk | No concern: High RoB in 3/7 studies | Downgrade: Results inconsistent | No concern | No concern | **Moderate** | Moderate evidence but Inconsistent associations between marital status and the risk of care home admission |
|  | **Living situation (alone, family, caregiver)** | 6 studies;  14 associations | 7 significant associations; Living alone 6 higher risk, 1 lower risk | No concern: High RoB in 1/6 studies | No concern | No concern | No concern | **High** | High evidence that living alone increases risk of care home admission |
|  | **Race** | 2 studies;  3 associations | 3 significant associations; white race higher risk | No concern:  No high RoB | No concern | No concern | No concern | **High** | High evidence that being white race increases risk of care home admission |
|  | **Migrant status** | 2 studies;  5 associations | 4 significant associations; native Dutch (vs western migrant) 2 studies higher risk, native Dutch /western migrant (vs non-western) higher 2 studies | No concern:  No high RoB | No concern | No concern | Downgrade: Data from the same cohort | **Moderate** | Moderate but limited evidence that being native Dutch (vs non-western) native or migrant increases risk of care home admission |
|  | **Deprivation** | 2 studies;  5 associations | 5 significant associations; less deprived higher risk | Downgrade: High RoB in 1/2 studies | No concern | No concern | No concern | **Moderate** | Moderate evidence that being less deprived increases risk of care home admission |
|  | **Income** | 1 study;  2 associations | 1 significant association; Lower income 1 study higher risk where not receiving any home care | No concern:  No high RoB | No concern | No concern | Downgrade: Only 1 study | **Moderate** | Moderate but limited evidence that lower income increases risk of care home admission |
|  | **Urban residence** | 2 studies;  2 associations | 2 significant associations; Higher risk for urban | Downgrade: High RoB in 1/2 studies | No concern | No concern | No concern | **Moderate** | Moderate evidence that residing in urban areas increases risk of care home admission |
|  | **Geographical region** | 1 study;  1 association | 1 significant association; Quebec, Prairies, British Columbia (vs Atlantic, Ontario) higher risk | No concern:  No high RoB | No concern | No concern | Downgrade: Only 1 study | **Moderate** | Moderate but limited evidence that residing in certain Canadian geographical regions increases risk of care home admission |
|  | **Type of residence** | 1 study;  3 associations | 2 significant associations; retirement/assisted living higher risk | No concern:  No high RoB | No concern | No concern | Downgrade: Only 1 study | **Moderate** | Moderate but limited evidence that living in a retirement or assisted living facility increases risk of care home admission |
| **General health & lifestyle** | **Alcohol** | 1 study;  2 associations | 0 significant associations | No concern:  No high RoB | No concern | No concern | Downgrade: Only 1 study | **Moderate** | Moderate but limited evidence that alcohol does not increase risk of care home admission |
|  | **BMI/weight** | 2 studies;  5 associations | 0 significant associations | Downgrade: High RoB in 1/2 studies | No concern | No concern | No concern | **Moderate** | Moderate evidence that bodyweight does not increase risk of care home admission |
|  | **Frailty** | 2 studies;  5 associations | 5 significant associations; 3 higher, 2 lower risk | Downgrade: High RoB in 1/2 studies | Downgrade: Results inconsistent | No concern | No concern | **Low** | Low evidence but Inconsistent associations between frailty and the risk of care home admission |
|  | **Self-reported general health** | 3 studies;  8 associations | 0 significant associations | No concern:  No high RoB | No concern | No concern | No concern | **High** | High evidence that self-rated health does not increase risk of care home admission |
|  | **Smoking** | 1 study;  1 association | 0 significant associations | No concern:  No high RoB | No concern | No concern | Downgrade: Only 1 study | **Moderate** | Moderate but limited evidence that smoking does not increase risk of care home admission |
| **Functional status** | **Activities of daily living** | 7 studies;  20 associations | 9 significant associations;  4 higher, 2 lower, 3 not stated | No concern: High RoB in 2/7 studies | Downgrade: Variation in measures and results inconsistent | No concern | No concern | **Moderate** | Moderate evidence but Inconsistent associations between activity of daily living and the risk of care home admission |
|  | **Mobility** | 5 studies;  7 associations | 4 significant associations;  3 higher, 1 lower risk | Downgrade: High RoB in 3/5 | Downgrade: Variation in measures and results inconsistent | No concern | No concern | **Low** | Low evidence but inconsistent associations between mobility and the risk of care home admission |
|  | **Falls/gait problems** | 1 study;  2 associations | 0 significant associations | No concern: No high RoB | No concern | No concern | Downgrade: Only 1 study | **Moderate** | Moderate but limited evidence falls/gait problems do not increase risk of care home admission |
|  | **Inactivity** | 1 study;  1 association | 0 significant associations | No concern: No high RoB | No concern | No concern | Downgrade: Only 1 study | **Moderate** | Moderate but limited evidence inactivity does not increase risk of care home admission |
|  | **Safety concerns** | 1 study;  2 associations | 0 significant associations | No concern: No high RoB | No concern | No concern | Downgrade: Only 1 study | **Moderate** | Moderate but limited evidence safety concerns do not increase risk of care home admission |
| **Social** | **Social withdrawal** | 1 study;  1 association | 0 significant associations | No concern: No high RoB | No concern | No concern | Downgrade: Only 1 study | **Moderate** | Moderate but limited evidence social withdrawal does not increase risk of care home admission |
|  | **Lonely** | 1 study;  4 associations | 0 significant associations | No concern: No high RoB | No concern | No concern | Downgrade: Only 1 study | **Moderate** | Moderate but limited evidence loneliness does not increase risk of care home admission |
| **Dementia characteristics** | **Cognition at baseline** | 10 studies;  21 associations | 11 significant associations; Higher risk | Downgrade: High RoB in 5/10 studies | Downgrade: Variation in measures & results inconsistent | No concern | No concern | **Low** | Low evidence that worse cognition at baseline increases risk of care home admission |
|  | **Type of dementia** | 2 studies;  2 associations | 1 significant associations; Higher risk | Downgrade: High RoB in 1/2 studies | Downgrade: Variation in measures | No concern | No concern | **Low** | Low evidence but inconsistent associations between type of dementia and risk of care home admission |
|  | **Taking dementia medication** | 4 studies;  6 associations | 4 significant associations; 3 higher, 1 lower rsk | No concern:  No high RoB | Downgrade: Variation in measures & results inconsistent | No concern | Downgrade: Data from same cohort for 4/6 associations | **Low** | Low evidence of taking dementia medication and increased risk of care home admission |
|  | **Duration of dementia** | 1 study;  1 association | 0 significant associations | Downgrade: High RoB in 1/1 studies | No concern | No concern | Downgrade: Only 1 study | **Low** | Low but limited evidence that duration of dementia increases risk of care home admission |
|  | **Difficulty understanding** | 1 study;  1 association | 0 significant associations | Downgrade: High RoB in 1/1 studies | No concern | No concern | Downgrade: Only 1 study | **Low** | Low but limited evidence that difficulty understanding increases risk of care home admission |
|  | **Difficulties communicating** | 1 study;  1 association | 0 significant associations | Downgrade: High RoB in 1/1 studies | No concern | No concern | Downgrade: Only 1 study | **Low** | Low but limited evidence that difficulty communicating increases risk of care home admission |
| **Neuropsychiatric** | **Mental health (composite)** | 2 studies;  4 associations | 0 significant associations | Downgrade: High RoB in 1/2 studies | No concern | No concern | No concern | **Moderate** | Moderate evidence mental health does not increase risk of care home admission |
|  | **Psychosis/ psychotic symptoms** | 4 studies;  6 associations | 3 significant associations;  Higher risk | No concern: High RoB 1/4 studies | No concern | No concern | No concern | **High** | High evidence that psychosis/ psychotic symptoms increase risk of care home admission |
|  | **Depression** | 4 studies;  5 associations | 3 significant associations; Higher risk | Downgrade: High RoB in 2/4 studies | No concern | No concern | No concern | **Moderate** | Moderate evidence that depression increases risk of care home admission |
|  | **Wandering** | 3 studies;  6 associations | 5 significant associations; Higher risk | Downgrade: High RoB in 2/3 studies | Downgrade: Variation in measures | No concern | No concern | **Low** | Low evidence that wandering increases risk of care home admission |
|  | **Agitation** | 2 studies;  2 associations | 0 significant associations | Downgrade: High RoB in 1/2 studies | No concern | No concern | No concern | **Moderate** | Moderate evidence agitation does not increase risk of care home admission |
|  | **Behavioural change** | 2 studies;  3 associations | 1 significant association; higher risk | Downgrade: High RoB in 1/2 studies | No concern | No concern | No concern | **Moderate** | Moderate evidence but inconsistent associations between behavioural change and the risk of care home admission |
|  | **Aggression** | 2 studies;  4 associations | 0 significant associations | Downgrade: High RoB in 1/2 studies | No concern | No concern | No concern | **Moderate** | Moderate evidence aggression does not increase risk of care home admission |
|  | **Anxiety** | 1 study;  1 association | 1 significant association; higher risk | No concern: No high RoB | No concern | No concern | Downgrade: Only 1 study | **Moderate** | Moderate but limited evidence anxiety increases risk of care home admission |
|  | **Tearful** | 1 study;  1 association | 0 significant associations | No concern: No high RoB | No concern | No concern | Downgrade: Only 1 study | **Moderate** | Moderate but limited evidence tearful/crying does not increase risk of care home admission |
|  | **Major stress** | 1 study;  1 association | 0 significant associations | No concern: No high RoB | No concern | No concern | Downgrade: Only 1 study | **Moderate** | Moderate but limited evidence major stress does not increase risk of care home admission |
|  | **Apathy** | 1 study;  1 association | 0 significant associations | Downgrade: High RoB in 1/1 studies | No concern | No concern | Downgrade: Only 1 study | **Low** | Low but limited evidence apathy does not increase risk of care home admission |
|  | **Irritable/easy to anger** | 1 study;  1 association | 1 significant association; Higher risk | No concern: No high RoB | No concern | No concern | Downgrade: Only 1 study | **Moderate** | Moderate but limited evidence irritable/easy to anger increases risk of care home admission |
|  | **Making accusations** | 1 study;  1 association | 0 significant associations | Downgrade: High RoB in 1/1 studies | No concern | No concern | Downgrade: Only 1 study | **Low** | Low but limited evidence making accusations does not increase risk of care home admission |
|  | **Fearful** | 1 study;  1 association | 0 significant associations | No concern: No high RoB | No concern | No concern | Downgrade: Only 1 study | **Moderate** | Moderate but limited evidence fearful does not increase risk of care home admission |
|  | **Orientation problems at home** | 1 study;  1 association | 1 significant association; Higher risk | Downgrade: High RoB in 1/1 studies | No concern | No concern | Downgrade: Only 1 study | **Low** | Low but limited evidence orientation problems at home increases risk of care home admission |
|  | **Difficulty going to bed** | 1 study;  1 association | 0 significant associations | Downgrade: High RoB in 1/1 studies | No concern | No concern | Downgrade: Only 1 study | **Low** | Low but limited evidence difficulty bringing to bed does not increase risk of care home admission |
|  | **Sleep disorders/ disturbance** | 1 study;  1 association | 0 significant associations | Downgrade: High RoB in 1/1 studies | No concern | No concern | Downgrade: Only 1 study | **Low** | Low but limited evidence sleep disorders/disturbance does not increase risk of care home admission |
|  | **Night-time activity** | 1 study;  1 association | 1 significant association; Higher risk | Downgrade: High RoB in 1/1 studies | No concern | No concern | Downgrade: Only 1 study | **Low** | Low but limited evidence night-time activity increases risk of care home admission |
|  | **Inappropriate urination** | 1 study;  1 association | 0 significant associations | Downgrade: High RoB in 1/1 studies | No concern | No concern | Downgrade: Only 1 study | **Low** | Low but limited evidence inappropriate urination does not increase risk of care home admission |
| **Symptoms** | **Bowel incontinence** | 3 studies;  7 associations | 1 significant association; Lower risk | Downgrade: High RoB in 2/3 studies | No concern | No concern | No concern | **Moderate** | Moderate evidence that bowel incontinence does not increase risk of care home admission |
|  | **Urinary incontinence** | 4 studies;  8 associations | No significant associations | Downgrade: High RoB in 2/4 studies | No concern | No concern | No concern | **Moderate** | Moderate evidence that urinary incontinence does not increase risk of care home admission |
|  | **Hearing loss** | 2 studies;  4 associations | No significant associations | Downgrade: High RoB in 1/2 studies | No concern | No concern | No concern | **Moderate** | Moderate evidence that hearing loss does not increase risk of care home admission |
|  | **Visual impairment** | 2 studies;  4 associations | No significant associations | Downgrade: High RoB in 1/2 studies | No concern | No concern | No concern | **Moderate** | Moderate evidence that visual impairment does not increase risk of care home admission |
|  | **Dyspnoea** | 1 study;  3 associations | 2 significant associations; Lower risk | No concern: No high RoB | No concern | No concern | Downgrade: Only 1 study | **Moderate** | Moderate but limited evidence dyspnoea decreases risk of care home admission |
|  | **Eating problems** | 1 study;  1 association | 0 significant associations | Downgrade: High RoB in 1/1 studies | No concern | No concern | Downgrade: Only 1 study | **Low** | Low but limited evidence eating problems does not increase risk of care home admission |
|  | **Extrapyramidal signs** | 1 study;  1 association | 1 significant association; Higher risk | Downgrade: High RoB in 1/1 studies | No concern | No concern | Downgrade: Only 1 study | **Low** | Low but limited evidence extrapyramidal signs increase risk of care home admission |
|  | **Fatigue** | 1 study;  3 associations | 2 significant associations; Lower risk | No concern: No high RoB | No concern | No concern | Downgrade: Only 1 study | **Moderate** | Moderate but limited evidence fatigue decreases risk of care home admission |
| **Comorbidities** | **No. of comorbidities** | 3 studies;  5 associations | 1 significant association; Higher risk | No concern: High RoB in 1/3 studies | No concern | No concern | No concern | **Moderate** | Moderate but inconsistent associations between the number of comorbid conditions and the risk of care home admission |
|  | **Hospitalisation** | 3 studies;  6 associations | 4 significant associations;  3 lower, 1 higher risk | No concern: No high RoB studies | Downgrade: Variation in measures | No concern | No concern | **Moderate** | Moderate evidence but inconsistent associations between hospitalisation and the risk of care home admission |
|  | **Cancer** | 4 studies;  5 associations | 2 significant associations;  Lower risk | No concern: No high RoB | No concern | No concern | No concern | **High** | High evidence that cancer does not increase risk of care home admission |
|  | **Cardiovascular disease** | 4 studies;  8 associations | 4 significant associations;  3 higher, 1 lower risk | No concern: No high RoB | Downgrade: Variation in conditions included & results inconsistent | No concern | No concern | **Moderate** | Moderate evidence but Inconsistent associations between cardiovascular disease and the risk of care home admission |
|  | **Cerebrovascular disease (inc. Stroke/TIA)** | 5 studies;  6 associations | 2 significant associations;  Higher risk | No concern: No high RoB | Downgrade: Results inconsistent | No concern | No concern | **Moderate** | Moderate but inconsistent associations between cerebrovascular disease and the risk of care home admission |
|  | **Diabetes** | 4 studies;  5 associations | 3 significant associations;  2 higher, 1 lower risk | No concern: No high RoB | Downgrade: Results inconsistent | No concern | No concern | **Moderate** | Moderate evidence but inconsistent associations between diabetes and the risk of care home admission |
|  | **Respiratory disease** | 3 studies;  3 associations | 1 significant association;  Lower risk | No concern: No high RoB | No concern | No concern | No concern | **High** | High evidence that respiratory disease does not increase risk of care home admission |
|  | **No. of doctor visits** | 1 study;  1 association | 0 significant associations | No concern: No high RoB | No concern | No concern | Downgrade: Only 1 study | **Moderate** | Moderate but limited evidence number of doctor visits does not increase risk of care home admission |
|  | **Hypercholesterolemia** | 1 study;  1 association | 0 significant associations | No concern: No high RoB | No concern | No concern | Downgrade: Only 1 study | **Moderate** | Moderate but limited evidence hypercholesterolaemia does not increase risk of care home admission |
|  | **Hypertension** | 1 study;  1 association | 0 significant associations | No concern: No high RoB | No concern | No concern | Downgrade: Only 1 study | **Moderate** | Moderate but limited evidence hypertension does not increase risk of care home admission |
|  | **Musculoskeletal disease** | 2 studies;  2 associations | 0 significant associations | No concern: No high RoB | No concern | No concern | No concern | **High** | High evidence musculoskeletal disease does not increase risk of care home admission |
|  | **Peptic ulcer disease** | 1 study;  1 association | 0 significant associations | No concern: No high RoB | No concern | No concern | Downgrade: Only 1 study | **Moderate** | Moderate but limited evidence peptic ulcer disease does not increase risk of care home admission |
|  | **Liver disease** | 1 study;  2 associations | 1 significant association; Mild disease lower risk | No concern: No high RoB | No concern | No concern | Downgrade: Only 1 study | **Moderate** | Moderate but limited evidence liver disease does not increase risk of care home admission |
|  | **Renal disease** | 1 study;  1 association | 0 significant associations | No concern: No high RoB | No concern | No concern | Downgrade: Only 1 study | **Moderate** | Moderate but limited evidence renal disease does not increase risk of care home admission |
| **Medications** | **Polypharmacy** | 4 studies;  6 associations | 1 significant association;  Lower risk | No concern: High RoB in 1 study | No concern | No concern | Downgrade: Data from same cohort for 4/6 associations | **Moderate** | Moderate evidence that polypharmacy does not increase risk of care home admission |
|  | **Antipsychotic medication** | 1 study;  6 associations | 5 significant associations;  Higher risk | No concern: No high RoB | No concern | No concern | Downgrade: Only 1 study | **Moderate** | Moderate but limited evidence that antipsychotics increase risk of care home admission |
|  | **Painkillers** | 1 study;  1 association | 1 significant association; Higher risk | Downgrade: High RoB in 1/1 studies | No concern | No concern | Downgrade: Only 1 study | **Low** | Low but limited evidence painkillers increase the risk of care home admission |
| **Caregiver and support** | **Currently receives care** | 6 studies;  19 associations | 11 significant associations; 7 higher risk, 4 lower | No concern: High RoB in 1/6 studies | Downgrade: Variation in measures | No concern | No concern | **Moderate** | Moderate evidence but Inconsistent associations between receiving care and the risk of care home admission |
|  | **Caregiver desire for admission** | 3 studies;  6 associations | 4 significant associations; Higher risk | No concern: High RoB in 1/3 studies | No concern | No concern | No concern | **High** | High evidence that caregivers desire for admission increases risk of care home admission |
|  | **Caregiver age** | 3 studies;  5 associations | 1 significant association; Older age higher risk | No concern: High RoB in 1/3 studies | Downgrade: Results inconsistent | No concern | No concern | **Moderate** | Moderate evidence but inconsistent associations of caregiver’s age with care home admission |
|  | **Caregiver sex** | 3 studies;  5 associations | 1 significant association; Female sex higher risk | Downgrade: High RoB in 2/3 studies | Downgrade: Results inconsistent | No concern | No concern | **Moderate** | Low evidence but inconsistent associations of caregiver’s sex with care home admission |
|  | **Caregiver burden/impact** | 1 study;  1 association | 1 significant associatios: higher burden higher risk | Downgrade: High RoB in 1/1 studies | No concern | No concern | Downgrade: Only 1 study | **Low** | Low but limited evidence that higher caregiver burden increases risk of care home admission |
|  | **Caregiver mental health** | 2 studies;  6 associations | 1 significant association; Higher risk | Downgrade: High RoB in 1/2 studies | No concern | No concern | No concern | **Moderate** | Moderate evidence that caregiver’s mental health does not increase risk of care home admission |
|  | **Caregiver physical health** | 2 studies;  4 associations | 1 significant association; More health problems higher risk | Downgrade: High RoB in 1/2 studies | Downgrade: Results inconsistent | No concern | No concern | **Low** | Low evidence but inconsistent evidence that caregivers' physical health is associated with care home admission |
|  | **Caregiver relationship** | 2 studies;  8 associations | 2 significant associations; Not spouse or child & child (vs spouse) higher risk | Downgrade: High RoB in 1/2 studies | Downgrade: Results inconsistent | No concern | No concern | **Low** | Low evidence but inconsistent evidence that caregiver's relationship is associated with care home admission |
|  | **Caregiver residence** | 2 studies;  7 associations | 0 significant associations | No concern: No high RoB | No concern | No concern | No concern | **High** | High evidence that caregiver’s residence does not increase risk of care home admission |
|  | **Caregiver quality of support** | 1 study;  9 associations | 1 significant association; Positive value of caregiving lowers risk | No concern: No high RoB | No concern | No concern | Downgrade: Only 1 study | **Moderate** | Moderate but limited evidence that higher caregiver quality of support does not increase risk of care home admission |
|  | **Duration of care** | 1 study;  3 associations | 1 significant association; longer care lowers risk in women | No concern: No high RoB | No concern | No concern | Downgrade: Only 1 study | **Moderate** | Moderate but limited evidence that duration of care does not increase risk of care home admission |
|  | **Size of support network** | 1 study;  1 association | 0 significant associations | Downgrade: High RoB in 1/1 studies | No concern | No concern | Downgrade: Only 1 study | **Low** | Low but limited evidence that size of support network does not increase risk of care home admission |
|  | **Caregiver difficulty taking responsibility** | 1 study;  1 association | 0 significant associations | Downgrade: High RoB in 1/1 studies | No concern | No concern | Downgrade: Only 1 study | **Low** | Low but limited evidence that caregiver difficulty taking responsibility does not increase risk of care home admission |
|  | **Caregiver family conflict** | 1 study;  1 association | 0 significant associations | Downgrade: High RoB in 1/1 studies | No concern | No concern | Downgrade: Only 1 study | **Low** | Low but limited evidence that caregiver family conflict does not increase risk of care home admission |
|  | **Caregiver guilt** | 1 study;  1 association | 0 significant associations | Downgrade: High RoB in 1/1 studies | No concern | No concern | Downgrade: Only 1 study | **Low** | Low but limited evidence that caregiver guilt does not increase risk of care home admission |
|  | **Caregiver employed** | 1 study;  1 association | 0 significant associations | Downgrade: High RoB in 1/1 studies | No concern | No concern | Downgrade: Only 1 study | **Low** | Low but limited evidence that caregiver employed does not increase risk of care home admission |
|  | **Caregiver not enough time for self** | 1 study;  1 association | 0 significant associations | Downgrade: High RoB in 1/1 studies | No concern | No concern | Downgrade: Only 1 study | **Low** | Low but limited evidence that caregiver not enough time for self does not increase risk of care home admission |
|  | **Caregiver loss of hobbies** | 1 study;  1 association | 0 significant associations | Downgrade: High RoB in 1/1 studies | No concern | No concern | Downgrade: Only 1 study | **Low** | Low but limited evidence that caregiver loss of hobbies does not increase risk of care home admission |
|  | **Caregiver sleep disturbance** | 1 study;  1 association | 0 significant associations | Downgrade: High RoB in 1/1 studies | No concern | No concern | Downgrade: Only 1 study | **Low** | Low but limited evidence that caregiver sleep disturbance does not increase risk of care home admission |
|  | **Caregiver main source of income** | 1 study;  1 association | 0 significant associations | Downgrade: High RoB in 1/1 studies | No concern | No concern | Downgrade: Only 1 study | **Low** | Low but limited evidence that caregiver main source of income does not increase risk of care home admission |

*^a^ number of studies is really number of papers as some papers use same study cohort;*

***^b^*** *Associations = total number of individual associations tested for this prognostic factor grouping – may be multiple associations extracted from an individual study.*

**Supplementary Table 7. GRADE assessments: summary of results for cognitive decline outcome**

Total number of studies = 26; 60 factors, total sample size = 17,116.

|  | **Prognostic Factor** | **Number of studies^a^ and associations^b^** | **Factor associations** | **Risk of Bias (RoB) summary** | **Consistency** | **Directness** | **Precision** | **Final GRADE** | **Factor summary** |  |
| --- | --- | --- | --- | --- | --- | --- | --- | --- | --- | --- |
| **Sociodemographic** | **Age** | 17 studies;  25 associations | 9 significant associations; 5 higher risk for older age, 4 higher risk for younger age | Downgrade:  High RoB in 12/17 studies. | Downgrade: Results inconsistent | No concern | No concerns | **Low** | Low evidence but inconsistent associations between older age and the risk of faster cognitive decline |  |
|  | **Sex** | 17 studies;  25 associations | 4 significant associations; 3 higher risk for females, 1 higher risk for males | Downgrade:  High RoB in 12/17 studies. | Downgrade: Results inconsistent | No concern | No concerns | **Low** | Low evidence but inconsistent associations between sex and the risk of faster cognitive decline |  |
|  | **Education** | 18 studies;  31 associations | 12 significant associations;  Higher risk for those with higher education (but better baseline) | Downgrade:  High RoB in 13/18 studies | Downgrade: Results inconsistent | No concern | No concerns | **Low** | Low evidence but inconsistent associations between education and risk of faster cognitive decline |  |
|  | **Race** | 1 study;  12 associations | 0 significant associations. | Downgrade:  High RoB in 1/1 studies. | No concern | No concern | Downgrade: Only 1 study | **Low** | Low but limited evidence that race is not associated with increased risk of faster cognitive decline |  |
|  | **Literacy** | 1 study;  1 association | 0 significant associations. | Downgrade:  High RoB in 1/1 studies. | No concern | No concern | Downgrade: Only 1 study | **Low** | Low but limited evidence that literacy is not associated with increased risk of faster cognitive decline |  |
|  | **Living situation** | 1 study;  2 associations | 2 significant associations; Higher risk for those living alone, Lower risk for living with family, Middle risk for nursing home | Downgrade:  High RoB in 1/1 studies | No concern | No concern | Downgrade: Only 1 study | **Low** | Low but limited evidence that living alone increases risk of faster cognitive decline |  |
|  | **Occupation** **(ever)** | 1 study;  1 association | 0 significant associations. | Downgrade:  High RoB in 1/1 studies. | No concern | No concern | Downgrade: Only 1 study | **Low** | Low but limited evidence that occupation not associated with increased risk of faster cognitive decline |  |
|  | **Urban residence** | 1 study;  1 association | 1 significant association; Higher risk in urban but better baseline | Downgrade:  High RoB in 1/1 studies. | No concern | No concern | Downgrade: Only 1 study | **Low** | Low but limited evidence that urban residence associated with increased risk of faster cognitive decline (but better baseline) |  |
| **General health & lifestyle** | **BMI** | 1 study;  1 association | 0 significant associations. | Downgrade:  High RoB in 1/1 studies | No concern | No concern | Downgrade: Only 1 study | **Low** | Low but limited evidence that higher BMI does not increase risk of faster cognitive decline |  |
|  | **Frailty** | 1 study;  1 association | 0 significant associations. | Downgrade:  High RoB in 1/1 studies | No concern | No concern | Downgrade: Only 1 study | **Low** | Low but limited evidence that frailty does not increase risk of faster cognitive decline |  |
|  | **Self-reported general health** | 2 studies;  2 associations | 0 significant associations. | Downgrade:  High RoB in 1/2 studies | No concern | No concern | No concern | **Moderate** | Moderate evidence that worse general health does not increase risk of faster cognitive decline |  |
|  | **Smoking** | 2 studies;  2 associations | 0 significant associations. | Downgrade:  High RoB in 1/2 studies | No concern | No concern | No concern | **Moderate** | Moderate evidence that smoking does not increase risk of faster cognitive decline |  |
| **Functional status** | **Activities of daily living** | 2 studies;  3 associations | 3 significant associations; Higher risk | Downgrade:  High RoB in 2/2 studies | No concern | No concern | Downgrade: Not included in multivariable analysis in 1 study | **Low** | Low but limited evidence that reduced activities of daily living increases risk of faster cognitive decline |  |
|  | **Falls/gait problems** | 1 study;  1 association | 0 significant associations. | Downgrade:  High RoB in 1/1 studies | No concern | No concern | Downgrade: Only 1 study | **Low** | Low but limited evidence that falls/gait problems do not increase risk of faster cognitive decline |  |
|  | **Gait speed** | 1 study;  12 association | 2 significant associations; Higher risk | Downgrade:  High RoB in 1/1 studies | No concern | No concern | Downgrade: Only 1 study | **Low** | Low but limited evidence that gait speed does not increase risk of faster cognitive decline |  |
|  | **Inactivity** | 1 study;  1 association | 0 significant associations. | Downgrade:  High RoB in 1/1 studies | No concern | No concern | Downgrade: Only 1 study | **Low** | Low but limited evidence that inactivity does not increase risk of faster cognitive decline |  |
|  | **Difficulty dressing** | 1 study;  1 association | 0 significant associations. | Downgrade:  High RoB in 1/1 studies | No concern | No concern | Downgrade: Only 1 study | **Low** | Low but limited evidence that difficulty dressing does not increase risk of faster cognitive decline |  |
| **Social** | **Social withdrawal** | 1 study;  1 association | 0 significant associations. | Downgrade:  High RoB in 1/1 studies | No concern | No concern | Downgrade: Only 1 study | **Low** | Low but limited evidence that social withdrawal does not increase risk of faster cognitive decline |  |
|  | **Social network** | 2 studies;  2 associations | 1 significant association; Poorer network higher risk | Downgrade:  High RoB in 2/2 studies | Downgrade: Results inconsistent | No concern | No concern | **Low** | Low but limited evidence that poorer social network increases risk of faster cognitive decline |  |
|  | **Type of dementia** | 3 studies;  5 associations | 3 significant associations; 2 Alzheimer’s has lower risk, 1 greater risk | Downgrade:  High RoB in 3/4 studies | Downgrade: Results inconsistent | No concern | No concern | **Low** | Low evidence but inconsistent associations between type of dementia and risk of faster cognitive decline |  |
|  | **Duration of dementia** | 5 studies;  6 associations | 3 significant associations; Higher risk for longer duration but 1 study it depends on activity level | Downgrade:  High RoB in 5/5 studies | No concern | No concern | Downgrade: 3 studies from same cohort | **Low** | Low evidence that duration of dementia associated with increased risk of faster cognitive decline |  |
|  | **Time from onset to diagnosis** | 1 study;  1 association | 1 significant association; Lower risk for longer time | No concern: No high RoB | No concern | No concern | Downgrade: Only 1 study | **Moderate** | Moderate but limited evidence that shorter time from onset to diagnosis increases risk of faster cognitive decline |  |
| **Dementia characteristics** | **Cognition** **at baseline** | 10 studies;  23 associations | 16 significant associations; 5 Higher risk in those with higher (better) baseline scores, 11 Higher risk for those with lower (worse) baseline scores | Downgrade:  High RoB in 7/9 studies | Downgrade: Variation in measures and results inconsistent | No concern | No concern | **Low** | Low evidence but inconsistent associations between baseline cognition and the risk of faster cognitive decline |  |
|  | **No. of cognitive activities** | 1 study;  1 association | 1 significant association; Higher risk in those engaging with fewer activities but greater in those with shorter disease duration | Downgrade:  High RoB in 1/1 studies | No concern | No concern | Downgrade: Only 1 study | **Low** | Low but limited evidence that engaging with fewer cognitive activities increases risk of faster cognitive decline |  |
|  | **Family history of dementia** | 1 study;  1 association | 0 significant associations. | Downgrade:  High RoB in 1/1 studies | No concern | No concern | Downgrade: Only 1 study | **Low** | Low but limited evidence that family history of dementia does not increase risk of faster cognitive decline |  |
| **Neuropsychiatric** | **Agitation/ aggression** | 3 studies;  3 associations | 3 significant associations; higher risk | No concern: High RoB in 1/3 studies | Downgrade: Variation in measures | No concern | No concern | **Moderate** | Moderate evidence that agitation and aggression increase risk of faster cognitive decline |  |
|  | **Apathy** | 2 studies;  2 associations | 0 significant associations | Downgrade:  High RoB in 1/2 studies | No concern | No concern | Downgrade: All data from the same cohort | **Low** | Low but limited evidence that apathy does not increase risk of faster cognitive decline |  |
|  | **Complaining** | 1 study;  1 association | 0 significant associations | Downgrade:  High RoB in 1/1 studies | No concern | No concern | Downgrade: only 1 study | **Low** | Low but limited evidence that complaining does not increase risk of faster cognitive decline |  |
|  | **Depression, anxiety & irritability** | 6 studies;  8 associations | 0 significant associations | Downgrade:  High RoB in 4/6 studies | Downgrade: Variation in measures | No concern | No concern | **Low** | Low evidence that depression, anxiety and irritability does not increase risk of faster cognitive decline |  |
|  | **No. of behavioural symptoms** | 2 studies;  2 association | 1 significant association;  Higher risk | Downgrade:  High RoB in 2/2 studies | Downgrade: Results inconsistent | No concern | No concern | **Low** | Low but limited evidence that behavioural symptoms increase the risk of faster cognitive decline |  |
|  | **Emotionally labile** | 1 study;  1 association | 0 significant associations. | Downgrade:  High RoB in 1/1 studies | No concern | No concern | Downgrade: Only 1 study | **Low** | Low but limited evidence that emotionally labile does not increase risk of faster cognitive decline |  |
|  | **Fearful** | 1 study;  1 association | 0 significant associations. | Downgrade:  High RoB in 1/1 studies | No concern | No concern | Downgrade: Only 1 study | **Low** | Low but limited evidence that being fearful does not increase risk of faster cognitive decline |  |
|  | **Wandering** | 1 study;  1 association | 0 significant associations. | Downgrade:  High RoB in 1/1 studies | No concern | No concern | Downgrade: Only 1 study | **Low** | Low but limited evidence that getting lost does not increase risk of faster cognitive decline |  |
|  | **Psychosis inc. hallucinations & paranoia** | 5 studies;  6 associations | 4 significant associations; Higher risk | Downgrade:  High RoB in 3/5 studies | No concern | No concern | No concern | **Moderate** | Moderate evidence that psychosis increases risk of faster cognitive decline |  |
|  | **Hoards things** | 1 study;  1 association | 0 significant associations. | Downgrade:  High RoB in 1/1 studies | No concern | No concern | Downgrade: Only 1 study | **Low** | Low evidence that hoarding things does not increase risk of faster cognitive decline |  |
|  | **Severe neuropsychiatric symptoms** | 1 study;  1 association | 1 significant association; Higher risk | No concern: No high RoB | No concern | No concern | Downgrade: Only 1 study | **Moderate** | Moderate but limited evidence that severe neuropsychiatric symptoms increases risk of faster cognitive decline |  |
|  | **Paces** | 1 study;  1 association | 0 significant associations. | Downgrade:  High RoB in 1/1 studies | No concern | No concern | Downgrade: Only 1 study | **Low** | Low but limited evidence that pacing does not increase risk of faster cognitive decline |  |
|  | **Sleep disorders/ disturbance** | 2 studies;  2 associations | 1 significant association; Higher risk | Downgrade:  High RoB in 1/2 studies | Downgrade: Results inconsistent | No concern | No concern | **Low** | Low but limited evidence that sleep disorders/disturbance increases risk of faster cognitive decline |  |
|  | **Tearful** | 1 study;  1 association | 0 significant associations. | Downgrade:  High RoB in 1/1 studies | No concern | No concern | Downgrade: Only 1 study | **Low** | Low but limited evidence that tearful does not increase risk of faster cognitive decline |  |
|  | **Activity disturbance** | 1 study;  1 association | 1 significant association; Higher risk | Downgrade:  High RoB in 1/1 studies | No concern | No concern | Downgrade: Only 1 study | **Low** | Low but limited evidence that activity disturbance increases risk of faster cognitive decline |  |
|  | **Difficulty with money** | 1 study;  2 associations | 0 significant associations. | Downgrade:  High RoB in 1/1 studies | No concern | No concern | Downgrade: Only 1 study | **Low** | Low but limited evidence that difficulty with money does not increase risk of faster cognitive decline |  |
| **Symptoms** | **Extrapyramidal signs** | 1 study;  1 association | 0 significant associations. | No concern: No high RoB | No concern | No concern | Downgrade: Only 1 study | **Moderate** | Moderate but limited evidence that extrapyramidal signs do not increase risk of faster cognitive decline |  |
|  | **Hearing impairment** | 1 study;  1 association | 0 significant associations. | Downgrade:  High RoB in 1/1 studies | No concern | No concern | Downgrade: Only 1 study | **Low** | Moderate but limited evidence that hearing impairment does not increase risk of faster cognitive decline |  |
|  | **Visual impairment** | 1 study;  1 association | 0 significant associations. | Downgrade:  High RoB in 1/1 studies | No concern | No concern | Downgrade: Only 1 study | **Low** | Moderate but limited evidence that using glasses does not increase risk of faster cognitive decline |  |
| **Comorbidities** | **No. of comorbidities** | 7 studies;  9 associations | 1 significant association; Higher risk | Downgrade:  High RoB in 6/7 studies | No concern | No concern | No concern | **Moderate** | Moderate evidence that increased no. of comorbidities does not increase risk of faster cognitive decline |  |
|  | **Cardiovascular** **disease** | 5 studies;  5 associations | 2 significant associations; Coronary heart disease Higher risk; Cardiovascular Lower risk (slower decline) | Downgrade:  High RoB in 3/5 studies | Downgrade: Results inconsistent | No concern | No concern | **Low** | Low evidence but inconsistent associations between cardiovascular disease and the risk of faster cognitive decline |  |
|  | **Hypercholesterolaemia** | 2 study;  2 associations | 1 significant association; Higher cholesterol higher risk | Downgrade:  High RoB in 1/2 studies | No concern | No concern | No concern | **Moderate** | Moderate evidence that increased cholesterol increases risk of faster cognitive decline |  |
|  | **Diabetes** | 3 studies;  3 associations | 1 significant association; Higher risk | Downgrade:  High RoB in 2/3 studies | Downgrade: Results inconsistent | No concern | No concern | **Low** | Low evidence but inconsistent associations between diabetes and risk of faster cognitive decline |  |
|  | **Hypertension** | 4 studies;  4 associations | 0 significant associations. | Downgrade: High RoB in 3/4 studies | No concern | No concern | No concern | **Moderate** | Moderate evidence that hypertension does not increase risk of faster cognitive decline |  |
|  | **Hypertension or stroke** | 1 study;  1 association | 1 significant association; Higher risk | No concern: No high RoB | No concern | No concern | Downgrade: Only 1 study | **Moderate** | Moderate but limited evidence that hypertension or stroke increases risk of faster cognitive decline |  |
|  | **Cerebrovascular disease (inc. Stroke/TIA**) | 3 studies;  3 associations | 0 significant associations. | Downgrade:  High RoB in 2/3 studies | No concern | No concern | No concern | **Moderate** | Moderate evidence that cerebrovascular disease does not increase risk of faster cognitive decline |  |
|  | **Musculoskeletal disease** | 1 study;  1 association | 0 significant associations. | Downgrade:  High RoB in 1/1 studies | No concern | No concern | Downgrade: Only 1 study | **Low** | Low but limited evidence that musculoskeletal disease does not increases risk of faster cognitive decline |  |
| **Medications** | **Anticholinergic medication** | 1 study;  1 association | 0 significant associations. | Downgrade:  High RoB in 1/1 studies | No concern | No concern | Downgrade: Only 1 study | **Low** | Low but limited evidence that anticholinergic medication do not increases risk of faster cognitive decline |  |
|  | **Antidepressant medication** | 1 study;  1 association | 0 significant associations. | Downgrade:  High RoB in 1/1 studies | No concern | No concern | Downgrade: Only 1 study | **Low** | Low but limited evidence that antidepressants do not increases risk of faster cognitive decline |  |
|  | **Antihypertensive medication** | 2 studies;  3 associations | 1 significant association;  Lower risk (slower decline but Alzheimer’s only) | Downgrade:  High RoB in 2/2 studies | No concern | No concern | No concern | **Moderate** | Moderate evidence that antihypertensives do not increase risk of faster cognitive decline |  |
|  | **Antipsychotic medication** | 1 study;  1 association | 1 significant association;  Higher risk | Downgrade:  High RoB in 1/1 studies | No concern | No concern | Downgrade: Only 1 study | **Low** | Low but limited evidence that antipsychotics increases risk of faster cognitive decline |  |
|  | **Diuretic medication** | 1 study;  1 association | 1 significant association;  Lower risk (slower decline) | Downgrade:  High RoB in 1/1 studies | No concern | No concern | Downgrade: Only 1 study | **Low** | Low but limited evidence that diuretics do not increase risk of faster cognitive decline |  |
|  | **NSAIDs** | 1 study;  1 association | 0 significant associations. | Downgrade:  High RoB in 1/1 studies | No concern | No concern | Downgrade: Only 1 study | **Low** | Low but limited evidence that NSAIDs do not increase risk of faster cognitive decline |  |
|  | **Sedative medication** | 1 study;  1 association | 0 significant associations. | Downgrade:  High RoB in 1/1 studies | No concern | No concern | Downgrade: Only 1 study | **Low** | Low but limited evidence that sedatives do not increase risk of faster cognitive decline |  |
|  | **No. of non-psychotics** | 1 study;  1 association | 0 significant associations. | Downgrade:  High RoB in 1/1 studies | No concern | No concern | Downgrade: Only 1 study | **Low** | Low but limited evidence that increased number of non-psychotics does not increase risk of faster cognitive decline |  |

*^a^ number of studies is really number of papers as some papers use same study cohort;*

***^b^*** *Associations = total number of individual associations tested for this prognostic factor grouping – may be multiple associations extracted from an individual study.*

## Supplementary Table 8. PRISMA 2020 checklist

| **Section and Topic** | **Item #** | **Checklist item** | **Location where item is reported** |
| --- | --- | --- | --- |
| **TITLE** | | |  |
| Title | 1 | Identify the report as a systematic review. | 1 |
| **ABSTRACT** | | |  |
| Abstract | 2 | See the PRISMA 2020 for Abstracts checklist. | 2 |
| **INTRODUCTION** | | |  |
| Rationale | 3 | Describe the rationale for the review in the context of existing knowledge. | 3-4 |
| Objectives | 4 | Provide an explicit statement of the objective(s) or question(s) the review addresses. | 4 |
| **METHODS** | | |  |
| Eligibility criteria | 5 | Specify the inclusion and exclusion criteria for the review and how studies were grouped for the syntheses. | 5-7 |
| Information sources | 6 | Specify all databases; registers; websites; organisations; reference lists and other sources searched or consulted to identify studies. Specify the date when each source was last searched or consulted. | 4-5 |
| Search strategy | 7 | Present the full search strategies for all databases; registers and websites; including any filters and limits used. | 4-5  Suppl Table 1 |
| Selection process | 8 | Specify the methods used to decide whether a study met the inclusion criteria of the review; including how many reviewers screened each record and each report retrieved; whether they worked independently; and if applicable; details of automation tools used in the process. | 5-7 |
| Data collection process | 9 | Specify the methods used to collect data from reports; including how many reviewers collected data from each report; whether they worked independently; any processes for obtaining or confirming data from study investigators; and if applicable; details of automation tools used in the process. | 6-7 |
| Data items | 10a | List and define all outcomes for which data were sought. Specify whether all results that were compatible with each outcome domain in each study were sought (e.g. for all measures; time points; analyses); and if not; the methods used to decide which results to collect. | 6-7 |
|  | 10b | List and define all other variables for which data were sought (e.g. participant and intervention characteristics; funding sources). Describe any assumptions made about any missing or unclear information. | 6-7 |
| Study risk of bias assessment | 11 | Specify the methods used to assess risk of bias in the included studies; including details of the tool(s) used; how many reviewers assessed each study and whether they worked independently; and if applicable; details of automation tools used in the process. | 7-8 |
| Effect measures | 12 | Specify for each outcome the effect measure(s) (e.g. risk ratio; mean difference) used in the synthesis or presentation of results. | 7-8 |
| Synthesis methods | 13a | Describe the processes used to decide which studies were eligible for each synthesis (e.g. tabulating the study intervention characteristics and comparing against the planned groups for each synthesis (item #5)). | 8 |
|  | 13b | Describe any methods required to prepare the data for presentation or synthesis; such as handling of missing summary statistics; or data conversions. | 8 |
|  | 13c | Describe any methods used to tabulate or visually display results of individual studies and syntheses. | 8 |
|  | 13d | Describe any methods used to synthesize results and provide a rationale for the choice(s). If meta-analysis was performed; describe the model(s); method(s) to identify the presence and extent of statistical heterogeneity; and software package(s) used. | 8 |
|  | 13e | Describe any methods used to explore possible causes of heterogeneity among study results (e.g. subgroup analysis; meta-regression). | 8 |
|  | 13f | Describe any sensitivity analyses conducted to assess robustness of the synthesized results. | 8 |
| Reporting bias assessment | 14 | Describe any methods used to assess risk of bias due to missing results in a synthesis (arising from reporting biases). | 8 |
| Certainty assessment | 15 | Describe any methods used to assess certainty (or confidence) in the body of evidence for an outcome. | 8 |
| **RESULTS** | | |  |
| Study selection | 16a | Describe the results of the search and selection process; from the number of records identified in the search to the number of studies included in the review; ideally using a flow diagram. | 10  Figure 1 |
|  | 16b | Cite studies that might appear to meet the inclusion criteria; but which were excluded; and explain why they were excluded. | N/A |
| Study characteristics | 17 | Cite each included study and present its characteristics. | Suppl Table 3 |
| Risk of bias in studies | 18 | Present assessments of risk of bias for each included study. | 10-11  Suppl Table 4 |
| Results of individual studies | 19 | For all outcomes; present; for each study: (a) summary statistics for each group (where appropriate) and (b) an effect estimate and its precision (e.g. confidence/credible interval); ideally using structured tables or plots. | 11-13 |
| Results of syntheses | 20a | For each synthesis; briefly summarise the characteristics and risk of bias among contributing studies. | 11-13  Suppl Tables 5 & 6 |
|  | 20b | Present results of all statistical syntheses conducted. If meta-analysis was done; present for each the summary estimate and its precision (e.g. confidence/credible interval) and measures of statistical heterogeneity. If comparing groups; describe the direction of the effect. | n/a |
|  | 20c | Present results of all investigations of possible causes of heterogeneity among study results. | n/a |
|  | 20d | Present results of all sensitivity analyses conducted to assess the robustness of the synthesized results. | n/a |
| Reporting biases | 21 | Present assessments of risk of bias due to missing results (arising from reporting biases) for each synthesis assessed. | 10-11  Suppl Table 4 |
| Certainty of evidence | 22 | Present assessments of certainty (or confidence) in the body of evidence for each outcome assessed. | 11  Suppl Tables 5 & 6 |
| **DISCUSSION** | | |  |
| Discussion | 23a | Provide a general interpretation of the results in the context of other evidence. | 14-17 |
|  | 23b | Discuss any limitations of the evidence included in the review. | 16-17 |
|  | 23c | Discuss any limitations of the review processes used. | 16-17 |
|  | 23d | Discuss implications of the results for practice; policy; and future research. | 15-17 |
| **OTHER INFORMATION** | | |  |
| Registration and protocol | 24a | Provide registration information for the review; including register name and registration number; or state that the review was not registered. | 4 |
|  | 24b | Indicate where the review protocol can be accessed; or state that a protocol was not prepared. | 4 |
|  | 24c | Describe and explain any amendments to information provided at registration or in the protocol. | n/a |
| Support | 25 | Describe sources of financial or non-financial support for the review; and the role of the funders or sponsors in the review. | 19 |
| Competing interests | 26 | Declare any competing interests of review authors. | 19 |
| Availability of data; code and other materials | 27 | Report which of the following are publicly available and where they can be found: template data collection forms; data extracted from included studies; data used for all analyses; analytic code; any other materials used in the review. |  |

*From: Page MJ; McKenzie JE; Bossuyt PM; Boutron I; Hoffmann TC; Mulrow CD; et al. The PRISMA 2020 statement: an updated guideline for reporting systematic reviews. BMJ 2021;372:n71. doi: 10.1136/bmj.n71*

For more information; visit: <http://www.prisma-statement.org/>
